# Machine Learning for Polaritonic Chemistry: Accessing Chemical Kinetics

**DOI:** 10.1021/jacs.3c12829

**Published:** 2024-02-14

**Authors:** Christian Schäfer, Jakub Fojt, Eric Lindgren, Paul Erhart

**Affiliations:** † Department of Physics, Chalmers University of Technology, 412 96, Göteborg, Sweden; ‡ Department of Microtechnology and Nanoscience, MC2, Chalmers University of Technology, 412 96, Göteborg, Sweden

## Abstract

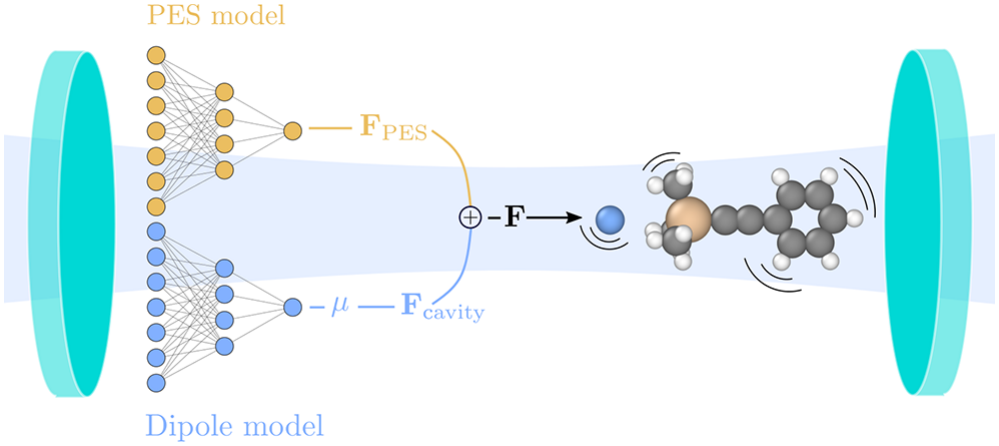

Altering chemical reactivity and material structure in confined optical environments is on the rise, and yet, a conclusive understanding of the microscopic mechanisms remains elusive. This originates mostly from the fact that accurately predicting vibrational and reactive dynamics for soluted ensembles of realistic molecules is no small endeavor, and adding (collective) strong light–matter interaction does not simplify matters. Here, we establish a framework based on a combination of machine learning (ML) models, trained using density-functional theory calculations and molecular dynamics to accelerate such simulations. We then apply this approach to evaluate strong coupling, changes in reaction rate constant, and their influence on enthalpy and entropy for the deprotection reaction of 1-phenyl-2-trimethylsilylacetylene, which has been studied previously both experimentally and using *ab initio* simulations. While we find qualitative agreement with critical experimental observations, especially with regard to the changes in kinetics, we also find differences in comparison with previous theoretical predictions. The features for which the ML-accelerated and *ab initio* simulations agree show the experimentally estimated kinetic behavior. Conflicting features indicate that a contribution of dynamic electronic polarization to the reaction process is more relevant than currently believed. Our work demonstrates the practical use of ML for polaritonic chemistry, discusses limitations of common approximations, and paves the way for a more holistic description of polaritonic chemistry.

## Introduction

If confined electromagnetic fields interact sufficiently strongly with matter, their excitations hybridize and give rise to new quasiparticles called polaritons.^1−6^ Strong light–matter coupling has been used to alter chemical reactivity,^7−13^ for which the term polaritonic chemistry has been coined. Vibrational strong coupling, in particular, is a promising candidate for practical application, demonstrating the inhibition,^9,14−16^ steering,^17^ and catalysis^18,19^ of chemical processes at room temperature. Especially appealing features include the ability to nonintrusively control the path of the chemical reaction by adjusting external parameters, such as the distance between mirrors, and its existence in the absence of any externally provided energy. The latter sets polaritonics apart from Floquet engineering, which typically suffers from heating and uncontrolled dissipation processes.^20^ Besides the specific control of chemical reactivity, polaritonics has been shown to give rise to a myriad of effects that range from commanding single molecules,^21−26^ over altering energy transfer,^27−43^ to the control of phase transitions in extended systems.^44−48^

Delivering a conclusive theoretical understanding for vibrational strong coupling has remained difficult. Especially, the experimentally observed resonance dependence in combination with an increase of rate changes for increasing emitter concentration and clear trends in chemical kinetics are critical features that a theoretical model should capture. Initial attempts based on the standard transition-state theory^49−51^ failed to reproduce any significant frequency dependence. Active development along the lines of Grote–Hynes^52−54^ and Pollak–Grabert–Hänggi^55^ theories showed some frequency dependence, but a connection to experiments has remained unsuccessful. A recent work by Schäfer et al.^13^ tackled the problem from first-principles by utilizing quantum electrodynamical density-functional theory (QEDFT)^56−59^ in combination with a self-consistent update of the nuclear motion according to Ehrenfest’s equation for the experimentally investigated deprotection reaction of 1-phenyl-2-trimethylsilylacetylene (PTA).^14,15^ This *ab initio* theory recovered critical components of the frequency dependence and suggested a microscopic theory based on adjusted vibrational energy redistribution for the cavity-induced modification of chemical reactivity. Chen et al. found experimental support for this hypothesis using 2D spectroscopy.^10^ Nonetheless, a prediction of kinetic quantities remained inaccessible, given the computational cost of QEDFT in combination with Ehrenfest dynamics.

In this work, we establish a framework that combines machine learning (ML) models, trained on data from density-functional theory (DFT) calculations, with molecular dynamics (MD) simulations to arrive at a more efficient, yet accurate description of the experimentally relevant S_N_2 reaction of PTA^14^ under strong coupling to a cavity. We find a pronounced frequency dependence for the chemical reaction rate constant along with changes in reaction enthalpy and entropy that are qualitatively consistent with the experiment. Interestingly, we discover frequency domains outside the experimentally validated window for which the present ML-accelerated approach predicts a rate constant enhancing character in contrast to earlier fully *ab initio* simulations,^13^ which rather suggest inhibition. Here, we tentatively attribute this difference to the simplifications inherent to the present MD approach, suggesting that the latter, despite being widely used, has relevant limitations in its applicability to polaritonic chemistry. Further investigations beyond the scope of the present work will be needed to provide a more detailed understanding.

## Methodology

Nonrelativistic quantum electrodynamics commonly starts at the minimal coupling Hamiltonian in the Coulomb gauge,^5,60−62^ where all charged particles couple via longitudinal Coulomb interaction to each other and to the transverse vector potential (∇·**A** = 0)
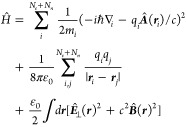
Polaritonic chemistry comes in different flavors that are distinguished by the frequency of the confined modes and the physical nature of the resonator, e.g., plasmonic, Fabry–Pérot, or whispering gallery cavities, which influences their fundamental coupling strength and quality factor. The accurate description of a realistic optical environment is a challenge in itself,^63−67^ but simple single-mode models often suffice to obtain a qualitative understanding of the relevant emitter dynamics.

The goal of this work is to provide a qualitative investigation of vibrational strong coupling and its influence on chemical reactivity for experimentally relevant molecules. In particular, we focus on kinetic changes, their impact on enthalpy and entropy, and the consequences of simplifications in the MD. For this reason, we stay conceptually close to the previous work of Schäfer et al.^13^ and rely on the Born–Oppenheimer approximation, projecting on the electronic ground-state and ignoring electronic polarizations induced by the cavity, such that the effective nuclear-photonic Hamiltonian takes the form^58,60,62,68^
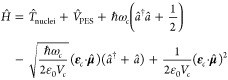
The nuclei couple self-consistently to a single electromagnetic mode of the cavity, treated classically as the mode displacement  in the following, with frequency ω_c_, effective cavity volume *V*_c_, fixed polarization ε_c_, and molecular dipole moment **μ̂**. Taking the classical limit for the nuclei, the system follows standard Hamiltonian mechanics with forces originating from the Poisson bracket . In order to see appreciable changes for the dynamics of a single molecule, we choose large coupling values  that are consistent with ref (13). We refer the interested reader to refs (13, 67, 69–71) for an extended discussion on the potential motivation of effective single-molecule coupling values. The chosen coupling is considerably larger than experimentally achievable values in Fabry–Pérot cavities, but the qualitative agreement with experiments suggests similar microscopic mechanisms. Our results and discussion can be partially transferred to plasmonic cavities which feature substantial single-molecule couplings.^21,23^

The *electronic* force acting on nucleus *j* is obtained from the potential energy surface (PES) according to
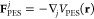
1and contributes to the total force **F**^*j*^ = **F**_PES_^*j*^ + **F**_c_^*j*^ together with the *optical* force, which can be computed from the derivative of the dipole moment vector
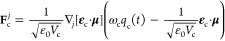
2The only interaction between light and matter arises then via the time evolution of the photonic mode *q*_c_(*t*) and the gradient of the projected dipole moment. The cavity mode displacement *q*_c_(*t*) depends on the history of the dipole moment (see Supporting Information Section IIA for details)
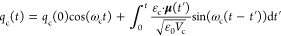
where vanishing of the initial momentum  is explicitly enforced.

We leverage the modular character of the forces by implementing a custom (“cavity”) propagator using the ase Python package.^72^ The calculator requires merely the initial molecular configuration as well as estimators for the forces arising from the PES (eq 1) and the dipole moment vector (eq 2). Possible estimators may include machine-learned models, empirical force fields, and even *ab initio* calculations based on, e.g., DFT. We note that this approach can also be easily combined with the embedding radiation-reaction approach^67^ in the future, thus providing an elegant path for including collective coupling and realistic optical environments.

Obtaining forces and dipole derivatives, thus, represents the main obstacle in the MD approach, as in reality, a molecule can easily pass through tens of thousands of configurations before a reaction occurs. One possible, but practically often too expensive, approach is to perform *ab initio* electronic structure calculations at each point, usually referred to as *ab initio* MD.^73^ While the cost of a single DFT calculation might be comparably low, the sheer quantity of calculations required for a statistically meaningful evaluation, i.e., obtaining thousands of trajectories with tens of thousands of DFT evaluations each, is highly prohibitive and practically limits the simulation time as well as the system and ensemble size. Another prominent approach is based on empirical force fields,^74,75^ which are computationally orders of magnitude more efficient than DFT calculations. They are, however, restricted with respect to accuracy as well as availability and offer limited transferability. Even if we would have a suitable force field for a system, there is no guarantee that, e.g., a force field fitted in an aqueous solution will perform well for simulations in vacuum and vice versa.

As we will show in the following, employing ML techniques in combination with MD provides a feasible, predictive, and scalable path, striking a balance between computational cost and accuracy.

### Electronic and Optical Forces Using Neural Networks

**Figure 1:**
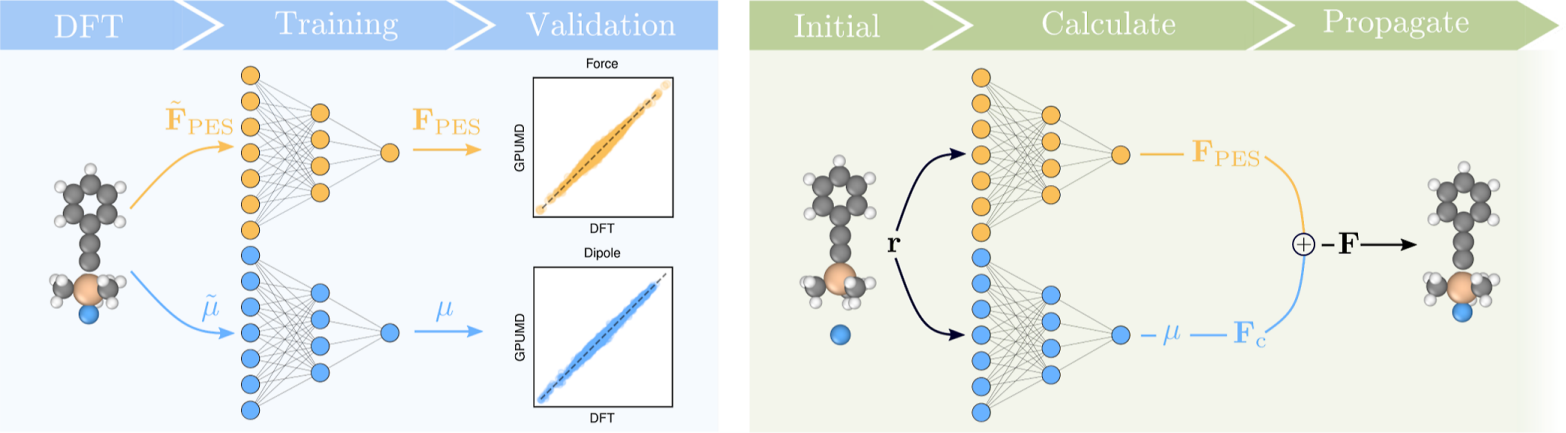
Methodological flow chart. DFT is used to calculate energies, forces, and dipoles. Positional information of a structure is translated into descriptor space, and NEP models for the PES and the dipole moment vector **μ** are trained using supervised learning (left-hand side). The final models are combined to compute the effective forces acting on the nuclei that are then used to propagate the system in time (right-hand side).

In the present work, we developed two different models using the neuroevolution potential (NEP) framework as implemented in the gpumd package.^76−78^ One for predicting the PES *V*_PES_(**r**), as well as the associated force **F**_PES_(**r**), and another for predicting dipole moments **μ**(**r**) (Figure 1; left). We refer the reader to Supporting Information for an extensive discussion of the training and testing procedures of both models.

The NEP models are then combined to obtain the total force used to propagate the system in time with a custom integrator implemented in the ase package^72^ (Figure 1; right). We refer the interested reader to refs (78 and 79) for a more extensive presentation of the NEP framework and its application to tensorial quantities.

The NEP approach employs a simple forward bias multilayer perceptron with a single hidden layer in combination with a flexible descriptor to predict the atomic energy *U*_*i*_ for each atom in a system by decomposing the total energy into individual contributions from each atom, *U* = ∑_*i*_*U*_*i*_. The model consists of a fully connected network with a single hidden layer, yielding the following expression for the predicted energy
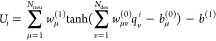
3The two weight matrices *w*_μ*v*_^(0)^ and *w*_μ_^(1)^ are the weights for the input and hidden layers, with *b*_μ_^(0)^ and *b*^(1)^ being their respective biases, and tanh is used as the activation function for the input layer. The so-called descriptor vector *q*_*v*_^*i*^ of length *N*_des_ indexed by *v* can be seen as a representation of the local chemical environment of atom *i*, is a function of the pairwise distances **r**_*ij*_ = **r**_*i*_ – **r**_*j*_, and serves as an input to the network. If *q*_*v*_^*i*^ uniquely describes a molecular configuration is determined by a basis expansion over *N*-body interactions within a cutoff radius *r*_c_. The expansion is truncated at 4-body interactions, which is sufficient to accurately describe local changes. A key feature of the NEP formalism is that these descriptors also contain trainable parameters, which allows the network to tailor the descriptors more individually to different atomic configurations. Predictions for forces and virials can be obtained by computing the gradient of the predicted site energies, i.e., the PES force acting on atom *i* is
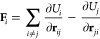
4Additionally, the NEP formalism may be extended to predict other tensorial properties, such as dipole moments, which are obtained as
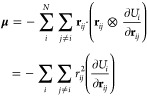
5

Having replaced the estimates for forces and dipoles with our NEP models, we are now equipped to address the question of how an optical resonator might influence the S_N_2 reaction.

## Results and Discussion

In a first step, we perform ensemble calculations with preserved particle number, volume, and temperature (*NVT*) in the absence of the cavity to obtain a reference value for the transition-state enthalpy of Δ*H*^‡^ = 0.345 eV = 33.3 kJ mol^–1^. The latter is in excellent agreement with experimental estimates of Δ*H*^‡^ = 35 ± 4 kJ mol^–1^.^15^ DFT calculations using the nudged elastic band approach in combination with a transition-state optimization provide a higher barrier of 0.43 eV. This illustrates the limitation of estimating the enthalpy from the 0 K energy difference between minimum and transition states and the significance of vibrational contributions. Detailed information as well as various benchmarks can be found in Supporting Information. We define a reaction event when the relevant Si–C bond is stretched beyond 3.5 Å, which exceeds the transition-state Si–C distance of approximately 3 Å, to avoid counting eventual recrossing events. If not further specified, all observables are obtained from ensemble averages involving 1000 trajectories. They are initialized with Boltzmann sampled velocities (kept fixed when changing cavity parameters) at the nonequilibrium F^–^ + PTA state used in ref (13) and propagated preserving particle number, volume, and energy (*NVE*). The cavity displacement is selected such that no electric field exists at time zero; i.e., the cavity force is zero. The initial state features an energy difference to the minimum PTAF^–^ configuration of 1.34 eV. This shortens the necessary calculation time and avoids spurious interplay between thermostat and cavity. One should note that it also limits the transferability of the obtained rate constants to experimental observations. Nonetheless, we can extract changes in the rate constant and thus contribute valuable insight into the current hypothesis behind polaritonic chemistry.

### Strong Coupling

**Figure 2:**
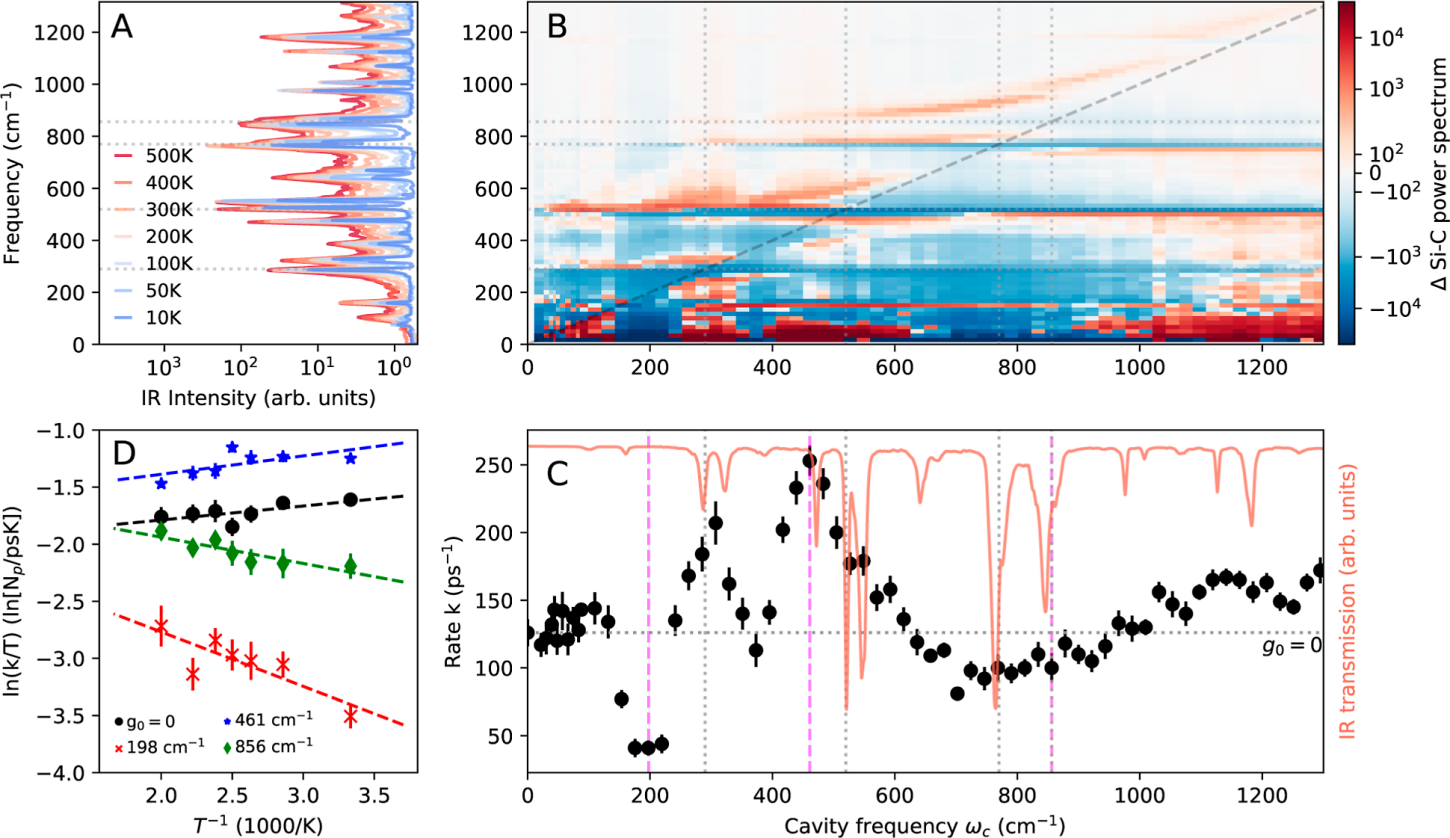
Modification of reaction rate constants by coupling to the cavity. (A) Temperature-dependent infrared spectrum outside the cavity, projected on the cavity polarization. (B) Difference in power spectra of the Si–C bond for PTAF^–^ coupled to the cavity with *g*_0_/ω_c_ = 1.132 at 400 K vs the same system outside the cavity. Gray-dotted lines (ℏω = 290, 520, 770, 856 cm^–1^) serve as guides to the eye. (C) Rate constant (black dots) and standard error (black bars) for the unidirectional reaction PTAF^–^ → FtMeSi + PA^–^ at 400 K and *g*_0_/ω_c_ = 1.132. The rate constant is calculated as number of products after 2 ps. Transmission spectrum at 400 K (red line, defined as negative of absorption spectrum). Vertical magenta-colored-dashed lines indicate relevant frequencies used for the kinetic estimates in (D). (D) Eyring plot in free space and for relevant cavity resonances given in inverse centimeter with *g*_0_/ω_c_ = 1.132. The extracted change in enthalpy and entropy is collected in Table 1. Negative enthalpy originates from the selected initial state and should only be interpreted relative to the equilibrium enthalpy (see text). The rate constant is calculated as number of products *N*_p_ per ps.

Strong coupling requires the existence of optically active vibrational modes near the cavity frequency. Certainly, the vibrational spectrum is sensitive to temperature, as illustrated in Figure 2A. The reaction-defining Si–C bond contributes fractionally to most vibrational excitations but primarily at frequencies below 1300 cm^–1^. Figure 2B illustrates the corresponding power spectrum during the reaction process at 400 K with strong coupling to the cavity. We keep the ratio *g*_0_/ω_c_ constant in our calculations. Changing the cavity length *L*_c_ (*V*_c_ = *A*_c_*L*_c_), which is the experimental way to tune the frequency of the Fabry–Pérot cavity, leads in a simplified mode picture to  and ω_c_ ∝ 1/*L*_c_, i.e., a larger distance between the mirrors reduces both frequency and coupling strength with 1/*L*_c_. Following the gray-dashed diagonal ω = ω_c_, we can clearly identify multiple avoided crossings (hybridizations) with substantial Si–C contribution. Each of the avoided crossings contributes with additional low-energy components (following the vertical gray-dotted lines), suggesting comparably slow changes, i.e., on the time scale of the reaction. The reorganization of the methyl groups and proper F–Si–C alignment (bending modes) are critical steps in the reaction pathway. Their interplay with the cavity manifests in the constant contribution at ℏω ≈ 117 cm^–1^, which is further detailed near the end of the manuscript.

### Resonance Dependence

Intuitively, we expect the low-frequency Si–C contribution to play a major role in the reactivity, as the defining Si–C bond-breaking step requires a few hundred femto seconds. Given the fact that the contribution changes nonmonotonically, it is not surprising that the rate constant also changes nonmonotonically (Figure 2C). We observe pronounced regions of inhibited reactivity, especially around 200 cm^–1^ and the domain including the 770 and 856 cm^–1^ vibrations. Furthermore, a near 2-fold enhancement of the rate constant at around 290 and 460 cm^–1^ is visible. Experimental investigations are available only near 856 cm^–1^ and support the general inhibiting trend in this domain, albeit featuring an inhibiting effect only in a narrow frequency window. A nonmonotonous rate change, however, is in conflict with previous *ab initio* simulations in ref (13). Interestingly, the most pronounced feature at 200 cm^–1^ seems to be not related to any optical excitation and is also not connected to the curvature at the transition state, which we estimate with both our NEP model and DFT calculations to be approximately 73 cm^–1^.

We will elaborate the conceptual differences to ref (13) and possible explanations later, but it should be noted that a strict comparison is difficult due to the difference in observable, temperature, and a substantial difference in statistical sampling. The slow but steady increase in the rate constant for large frequencies could partially originate from numerical deviations in the finite-difference approximation of the dipole gradient (see conservation of energy in Supporting Information). With this in mind, let us disentangle the mechanism behind the catalyzing and inhibiting effects by estimating enthalpic and entropic changes.

### Kinetics

We repeat calculations for the rate constant for various temperatures at four frequencies extracted from Figure 2C that are characteristic for their respective frequency domains. The results are collected in an Eyring plot (Figure 2D) and indicate conceptually different mechanisms for inhibition and rate constant enhancement. The corresponding Arrhenius plot is provided in Supporting Information. We would like to emphasize that those changes originate from the independent dynamics of an ensemble of trajectories and the Eyring equation
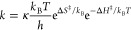
is used only to enable a comparison with the experimentally extracted enthalpy and entropy. Changes in transmission coefficient κ thus contribute to an altered entropy.

Without a cavity environment (ω_c_ = 0, black), we estimate a weak but negative enthalpic barrier Δ*H*. This should be seen as the change induced by the chosen initial state and *NVE* conditions, i.e., the elevated initial state will provide almost no energetic barrier, and yet, the cavity will alter this “barrier”. We demonstrate in Supporting Information Section IIB that performing rate constant estimates under *NVT* conditions and sufficient equilibration time provides accurate rates in agreement with the experiment outside the cavity, i.e., our methodology provides the correct kinetics outside the cavity, but the chosen initial state shifts the enthalpy up in energy. We collect the changes in enthalpy ΔΔ*H* and entropy ΔΔ*S* in relation to the cavity-free *NVE* results in Table 1.

**Table 1:** Change in Enthalpy and Entropy Compared to Free-Space Eyring Result (Black Data in Figure 2D)

ω_c_ (cm^–^^1^)	ΔΔ*H* (eV)	ΔΔ*S* (*k*_B_)
198	+0.052	+0.22
461	–0.003	+0.32
856	+0.030	+0.56

At the inhibiting frequencies of 198 cm^–1^ (red) and 856 cm^–1^ (green), the enthalpy Δ*H* increases considerably. The ω_c_ = 198 cm^–1^ excitation, without optically active vibrational mode support, shows a weak increase in entropy, suggesting that the dynamic effect of the cavity is to “simply” raise the transition-state barrier. Even though ω_c_ = 856 cm^–1^ (green) shows an overall smaller change, entropic changes are large compared to the other frequencies, which suggests that the cavity assigns a slightly stronger dissociative character to the reaction. Both features are in qualitative agreement with experiment.^14,15^ Taking into account that the correct enthalpy obtained from our *NVT* calculations in free space is Δ*H*^‡^ = 0.345 eV, the inhibiting frequencies render the reaction more temperature sensitive, which can lead to enhanced rates for large temperatures. Such a trend in temperature sensitivity has been widely observed in experiments.^14,15,18^

At the catalyzing frequency ω_c_ = 461 cm^–1^, on the other hand, there is almost no change in enthalpy, but there is a noticeable change in entropy, suggesting that the mechanism dominating here is not related to the experiments which typically showed a clear change in enthalpy. It is important to emphasize that utilization of the Eyring equation is especially problematic in this domain as a further increase in reaction speed implies that the trajectory will spend less time around the reactant well. This in turn implies that the kinetic arguments underlying the Eyring equation, i.e., a separation of time scales between reactant equilibration and transmission process, become questionable since the transition state is then part of the equilibration process. Nonetheless, a similar offset in the rate constant without change in enthalpy has been observed when employing Grote–Hynes rate theory.^80^ All three domains relate to different kinetic changes and, thus, suggest slightly different mechanisms.

### Vibrational Dynamics

**Figure 3:**
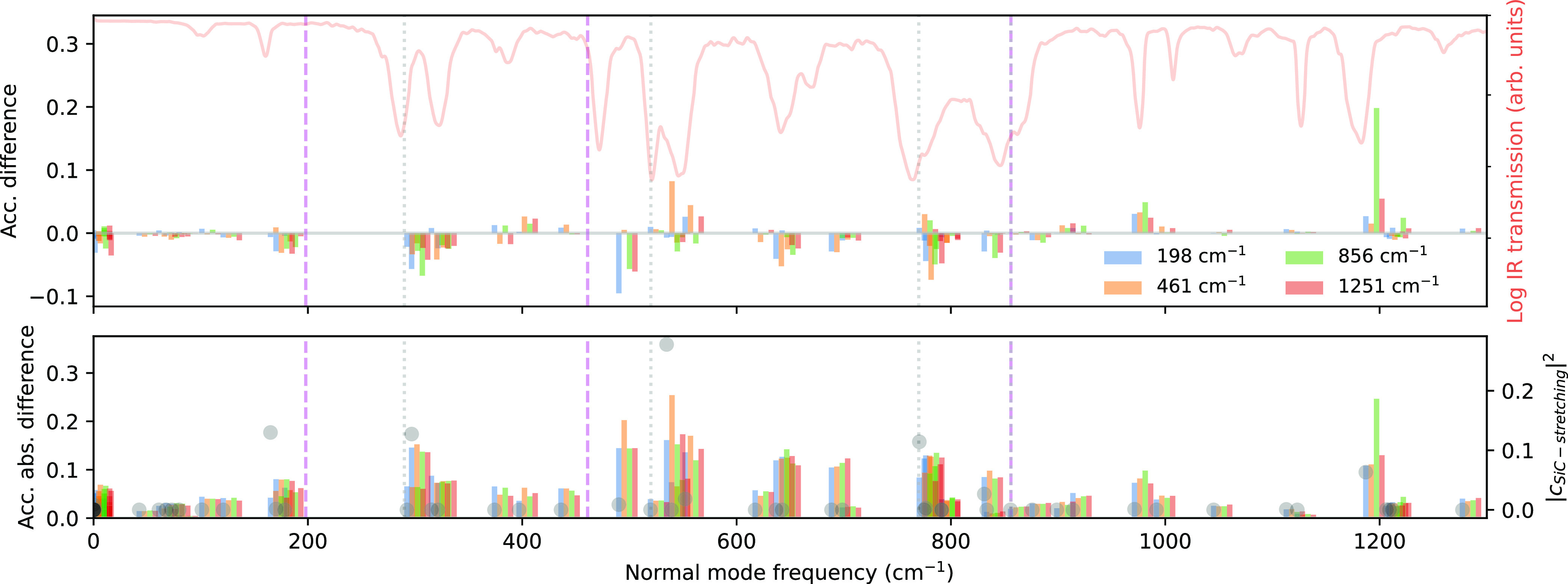
Change in mode occupation due to coupling to the cavity. Accumulated (top) and accumulated absolute (bottom) difference in the normal-mode occupation for different cavity frequencies (given in cm^–1^) with *g*_0_/ω_c_ = 1.132 vs free space at 400 K. The corresponding differences in occupation and numerical details are presented in the Supporting Information. We quantify the relative changes in cross-correlation between the IR spectrum and the accumulated (absolute) difference in Table 2. Gray circles show the contribution of Si–C stretching to the vibrational normal mode (see text and Supporting Information Section IIF). Note that we average here over a time domain of 2 ps and not 0.7 ps as in ref (13).

Let us shine a bit more light on the mechanistic differences between the chosen frequency domains that catalyze (461 cm^–1^) or inhibit reactions without (198 cm^–1^) or with (856 cm^–1^) vibrational support. Figure 3 illustrates the accumulated difference in normal-mode occupation between a given cavity frequency and free space during the reaction averaged over the full ensemble. The corresponding occupation differences are presented in the Supporting Information, where the overall structure for ω_c_ = 856 cm^–1^ is comparable to ref (13). Optically relevant domains around 300, 550, 770, and 1200 cm^–1^ are noticeably affected more strongly when selecting cavity frequencies ω_c_ = 461 cm^–1^ and ω_c_ = 856 cm^–1^. Choosing a specific cavity frequency affects the vibrational modes in energetic proximity more strongly. This effect is especially apparent for ω_c_ = 856 cm^–1^ which involves the C=C stretching mode around 1200 cm^–1^ (see the green bars in Figure 3). We quantify the changes in cross-correlation between the IR spectrum and the accumulated (absolute) difference [Figure 3 top (bottom)] in Table 2 with the help of the relative difference between the cross-correlation for ω_c_ ∈ {198, 461, 856} cm^–1^ and the high-frequency value ω_c_ = 1251 cm^–1^. The resulting change indicates how strongly differences in the normal-mode occupation correlate with infrared activity. Changes in the normal-mode occupation for cavity frequencies with optical support, i.e., ω_c_ = 461 cm^–1^ and ω_c_ = 856 cm^–1^, feature a larger correlation.

**Table 2:** Relative Difference of the Cross-Correlation Function a

ω_c_ (cm^–^^1^)	δδ*C*_ωc_^*AD*^ (%)	δδ*C*_ωc_^*AAD*^ (%)
198	+0.24	+1.09
461	+66.3	+11.3
856	+45.3	+3.11

a Only the frequency domain illustrated in Figure 3 was utilized in the calculation of the cross-correlation function. We define δ*C*_ω_c__ = ∑_*i*_|IR(ω_*i*_)·*A*(*A*)*D*_ω_c__(ω_*i*_)|/∑_*i*_IR(ω_*i*_), where *A*(*A*)*D* represents the accumulated (absolute) difference

Overall stronger correlation for ω_c_ = 461 and 856 cm^–1^ suggests that the microscopic mechanism is more strongly characterized by redistribution of vibrational energy between optically active modes and is thus optically mediated, as suggested in ref (13). In other words, the cavity facilitates energy exchange between optically active modes, especially within an energy window around the cavity frequency. Since the Si–C bond is an essential ingredient in the reaction process, a stronger involvement of the Si–C bond stretching (gray circles shown in Figure 3) in the affected normal modes will result in a larger impact on the chemical reaction. Then also, the vibrational analysis supports the previous hypothesis that chemical changes without support by optically active modes follow, to a certain degree, a different mechanism.^13^ Surprisingly, however, even though ω_c_ = 461 and 856 cm^–1^ seem to share a very similar mechanism based on the ML + MD analysis, their effect on the enthalpy is qualitatively different (catalyzing vs inhibiting). Albeit previous *ab initio* calculations did not provide access to kinetic changes, the corresponding analysis provided no indication of a qualitative difference between ω_c_ = 461 cm^–1^ and ω_c_ = 856 cm^–1^.^13^ Let us reflect in the following section on the underlying approximation of our MD simulations in order to understand this contradicting observation.

### Limitations of Simplified Cavity MD

The experimentally relevant domain around 856 cm^–1^^14,15^ is inhibited in our ML + MD calculations as well as in experiment and the recent *ab initio* QEDFT calculations with nuclear motion according to the Ehrenfest equations of motion.^13^ The existing information on changes in chemical rates is thus consistent at ω_c_ = 856 cm^–1^. On the other hand, the ML + MD calculations show enhanced rate constants at 461 cm^–1^, an effect that has not been observed in the QEDFT calculations. A direct comparison of Figure 2 to ref (13) is, however, problematic since the latter used an incomplete sampling with a strong bias toward the high-energy tail of the Boltzmann distribution. In other words, the QEDFT results were obtained at an effectively higher temperature.

**Figure 4:**
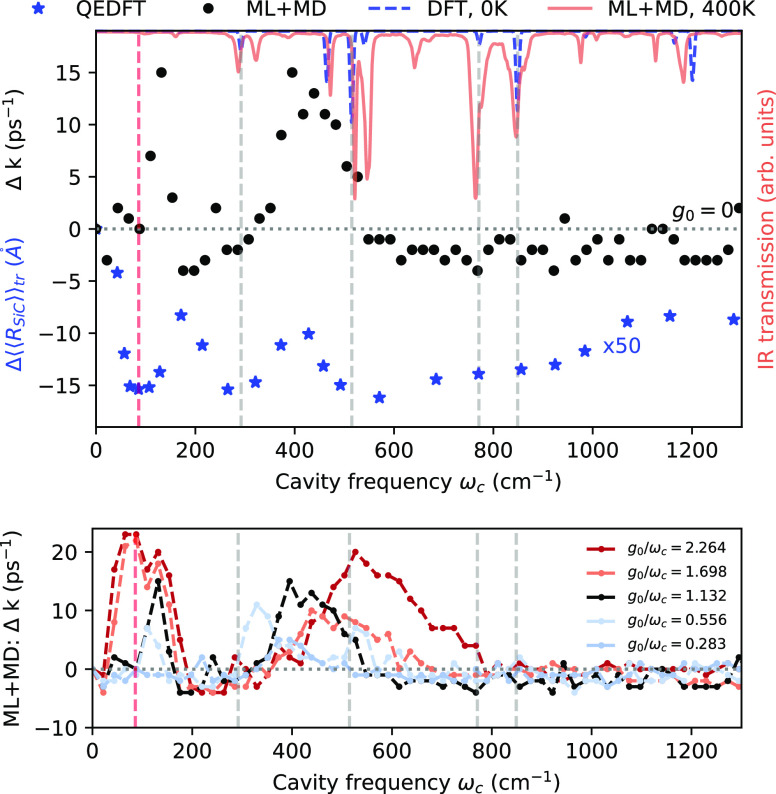
Impact of dynamic electronic polarization. (Top) Black dots: change in the rate constant compared to free space for the unidirectional reaction PTAF^–^ → FtMeSi + PA^–^ using the 30 initial configurations used in ref (13) and *g*_0_/ω_c_ = 1.132, but propagated with our NEP-based MD calculator. Blue stars: change in the average Si–C distance obtained from the QEDFT calculations in ref (13) amplified by a factor 50. Transmission spectrum obtained from DFT at 0 K with harmonic approximation consistent with ref (13) (blue dashed) and using our NEP model and GPUMD at 400 K *NVE* conditions (red solid). Vertical lines indicate characteristic features observed in ref (13). We show in Supporting Information Section IID that the Si–C distance and the rate constant are clearly correlated, i.e., the qualitative trend of QEDFT and ML + MD can be set in relation. (Bottom) ML + MD calculations repeated for different fundamental light–matter coupling strength.

To allow for a more reliable comparison and to shed light on the apparent discrepancy between the approaches, we recalculated the rate constant changes with our ML + MD approach for the same initial velocities as the QEDFT calculations. Figure 4 sets the newly obtained rate constants from our ML + MD calculations (black dots) in contrast with the average Si–C distances calculated with QEDFT (blue stars) and taken from ref (13). We demonstrate in Supporting Information Section IID that the Si–C distance and the rate constant are well correlated in this case.

Ignoring the offset, a slight frequency shift, and the qualitatively different behavior near ω_c_ = 0, the overall shapes of the reaction rate constant profiles shown in Figure 4 are consistent. Given identical initial conditions, ML + MD and QEDFT provide thus a similar *profile* in the intermediate frequency domain, but this profile is elevated into the catalyzing domain for the ML + MD calculations. Looking back at Figure 2C, the major catalyzing feature of the ML + MD calculations at 461 cm^–1^ is consistent between proper (Figure 2C) and incomplete sampling (Figure 4). Especially low-frequency features are considerably shifted and altered in strength, potentially originating from the quicker average reaction time when sampling from the high-energy tail of the Boltzmann distribution, which implies that the cavity has less time to influence the reaction. We recall that shorter reaction times emphasize dynamic contributions, calling the concept of a kinetic reaction rate further into question. It is thus plausible that the overwhelming catalyzing strength is partially an artifact, and we therefore suggest to focus on the qualitative trend only. This leaves but one question: Can we draw any conclusion about the mechanism of vibrational strong coupling from the observed discrepancy?

### Relevance of Dynamic Electronic Polarization

As we established at the beginning, effective nuclear forces comprise the adiabatic electronic Born–Oppenheimer forces and the dynamic optical forces mediated via dipolar changes induced by nuclear displacement. This implies that the static polarization of the electric system due to the instantaneous cavity field as well as its nonadiabatic corrections, quantum nuclear, and quantum light–matter effects is absent, as is the case in most available theoretical investigations for chemical reactivity affected by strong coupling. Nonadiabatic electron–nuclear effects are expected to play a minor role as the electronic excited space is separated by about 3 eV at minimum and transition states. While there has been recent discussions about the potential need to consider the full quantum light–matter interaction in model systems to recover resonant features in the cavity modified reactivity,^81,82^ it remains up to debate if this is true for realistic systems under standard ambient conditions. Such a question requires a nuanced discussion based on the specific system at hand, and we expect that the answer will vary strongly between collective and single molecular coupling.

Lastly, and probably most importantly, nuclear motion will induce strong optical fields, which in turn polarize the electronic system. This happens similar to a static external potential  or via the self-polarization contributions  (and ). It is important to note that this polarization, although being instantaneous in the sense of the Born–Oppenheimer approximation, is inherently *dynamic* due to its origin from nuclear dynamics. We will label this effect in the following *dynamic* electronic polarization to emphasize that the effect does not originate from the hybridization of the light–matter ground state nor from electronic transitions (nonadiabatic coupling elements). This form of dynamic electronic polarization can be formally incorporated via the cavity Born–Oppenheimer approximation,^83,84^ where the photonic displacement *q*_c_ is considered as an additional parametric variable and self-polarization contributions are added to the electronic structure calculations. When included, the dynamic electronic polarization can lead to notable asymmetries in the vibrational polaritons^13,84^ and increase the effective reaction barrier, thus reducing the chemical reactivity.^71,85^ Our current ML + MD calculations are lacking the possibility to describe this dynamic electronic polarization and will thus tend toward a higher reactivity and more symmetric Rabi splittings (see Figure 2B).

Consulting perturbation theory, the cavity induced changes in the electronic ground-state energy scale approximately as ∝*g*_0_^2^/ω_c_[1 – ω_c_/(Δ*E*_e_ + ω_c_)],^59,86^ where Δ*E*_e_ ≫ ω_c_ represents the electronic excitation energy for the dominant dipole transitions. Perturbation theory should provide an adequate estimate for electronic changes since the expansion parameter  is small and only *g*_0_/ω_c_ takes appreciable values. Fixing again *g*_0_/ω_c_ = const. and noticing a larger dipole near the transition state (see Note^a^), an additional increase of the barrier ∝ω_c_ would be missing in our MD calculations compared to the QEDFT calculations. As a result, our ML + MD calculations provide sensible values near ω_c_ = 0 and for large frequencies but have the tendency to be overly reactive in the intermediate domain as they lack an additional suppression from dynamic electronic polarization.

Larger cavity frequencies ω_c_ are driven resonantly only by vibrational modes of comparable frequency. If such a vibration contributes to the reaction, i.e., whether it will be noticeably excited during the reaction, depends largely on its Si–C contribution. If the cavity can exert notable effects on a reaction event will therefore depend on three time scales: (i) the strongly occupied reactive modes of frequency ω_SiC_, (ii) the cavity frequency ω_c_, and (iii) the frequency with which the PES is modulated ω_modPES_ (dynamic electronic polarization) as consequence of the nuclear dynamics. Since ω_modPES_ depends partially on the optical mode *q*_c_, which oscillates with ω_c_, it seems intuitive that the largest effect of dynamic electronic polarization on the reactive modes ω_SiC_ can be expected when all time scales are comparable. As emphasized by Figures 2B and 3, most of the optically active modes with relevant Si–C contributions are located below 840 cm^–1^. The *dynamic* contribution of electronic polarization then plays a decreasing role at higher frequencies. How the precise interplay between nuclear motion, strongly coupled cavity, and cavity-modulated electronic polarization affects the reactivity goes beyond the scope of this work. Our results emphasize the role of dynamic electronic polarization but also indicate that it is unlikely to be the only relevant contribution. Ample experimental work, however, demonstrated changes in solute–solvent interaction^18,88^ under vibrational strong coupling, which indicates the involvement of dispersive interactions mediated by electronic polarization.

### Additional Considerations

**Figure 5:**
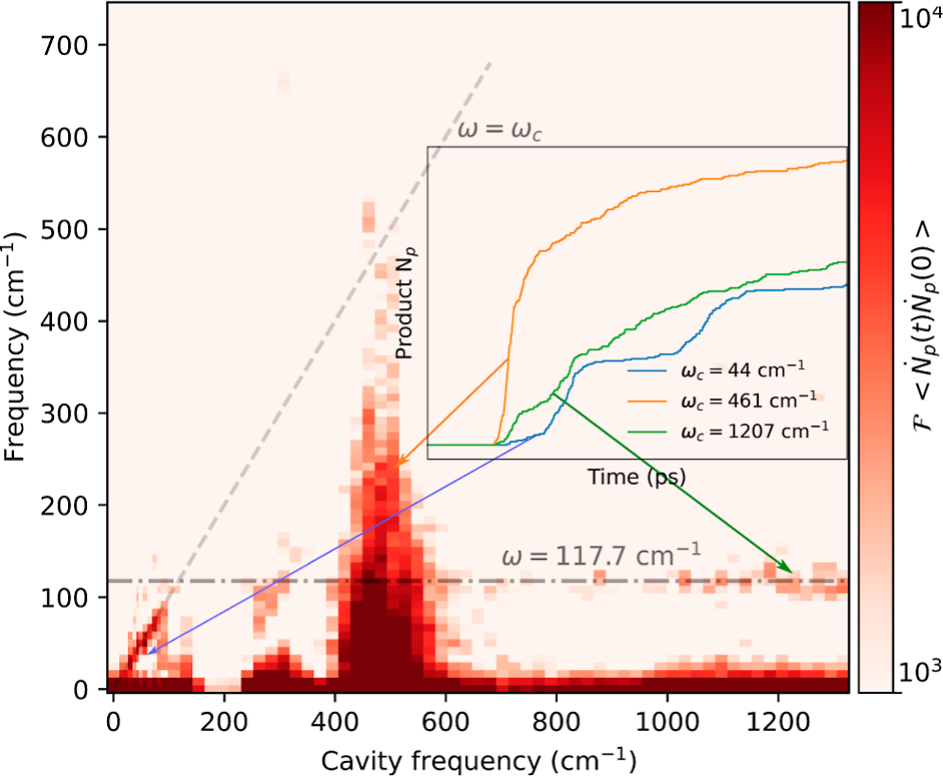
Frequency dependence of reaction bursts. Fourier transform of the autocorrelation function of changes in the number of products  at 400 K and *g*_0_/ω_c_ = 1.132, where *N*_p_ is the number of products. The inset shows the time-dependent accumulation of products at low frequencies, in the strongly catalyzing domain, and at high cavity frequencies where the 117 cm^–1^ mode attains a pronounced role during the reaction (see text).

Let us consider the analogy of our MD calculations as the self-consistently driven dynamic of a ball on a high-dimensional energy surface. If we intend to cross a specific barrier but let the cavity periodically remove kinetic energy before inserting it back at a later time, we can imagine that the likelihood to cross the barrier is modulated by the frequency with which the cavity is oscillating. We can indeed observe such an effect at low cavity frequencies, where reaction events appear in bursts that are related to the cavity frequency. Figure 5 shows the Fourier transform of the autocorrelation function of the change in product of the S_N_2 reaction. The linear dispersion at low frequencies clearly shows that the bursts in reaction events are correlated with the cavity frequency, a feature that is absent in the QEDFT calculations of ref (13). This trend continues up to a bending mode at 117 cm^–1^, which contributes to the necessary rearrangement of the methyl groups. Bending modes have recently been identified as a relevant component in the cavity-enhanced charge transfer,^38^ which might suggest that the observed interplay between linear dispersion and bending mode could be of wider relevance. Surely, this simplified picture of modulated reactivity can only hold if vibrational energy redistribution into other modes is unlikely, a condition that is rarely fulfilled and explains why the effect disappears quickly.

It seems intuitive to assume that the here-observed discrepancy, supposedly scaling with *g*_0_^2^, should only matter for sizable coupling strengths, and the change in the rate constant should approach the cavity-free reference value monotonously for decreasing coupling. However, this would either imply that the ML + MD calculation would need to qualitatively change from catalysis to inhibition while approaching , which is not observed in Figure 4 (bottom), or that the QEDFT calculations should become catalyzing during this process, for which there was no indication in ref (13). One might thus draw the conclusion that dynamic electronic polarization, missing in ML + MD, is required for the qualitative prediction of chemical changes via single-molecule vibrational strong coupling at most frequencies and for all coupling values. Furthermore, such features might even play a role in collectively coupled systems where individual molecules can exhibit large dynamic electronic polarization in response to collective vibrational dynamics.^70,71^

Supporting Information Section IIH includes the same investigation performed in this section using a second NEP model based on DFT data calculated with a smaller electronic basis-set. The overall trend up to 700 fs (as in ref (13)) is consistent with the here-presented ML + MD calculations. Since both ML + MD investigations show a consistent qualitative deviation from the QEDFT calculations, they both support the argument of dynamic electronic polarization. In addition, the smaller basis-set results in a smaller reaction barrier, which reduces the significance of the catalyzed trajectories at longer times. The most characteristic feature remaining after 2 ps is a strong inhibition around 200 cm^–1^, which is consistent with Figure 2C. This emphasizes that estimates of kinetic quantities require sufficient sampling.

Sampling, calculating, and learning potentials for every frequency and coupling, in order to obtain the full cavity Born–Oppenheimer surface, is in principle possible. If we intend to widely scan those parameters or would like to include more than a single mode, it becomes, however, practically unfeasible. A (self-consistent) perturbative correction^84,85,89^ to the electronic quantities might suffice to suppress some of the problematic features. Such a correction could be constructed via static electronic polarizabilities, which can be predicted with an additional neural network,^79^ to correct the electronic energies and dipole moments according to the adiabatic cavity field. We envision to combine such a corrected treatment with a full graphical processor unit (GPU) implementation of our approach, which would dramatically reduce the required computational time and pave the way toward explicit ensembles, solvents, and accurate estimates of the chemical kinetics.

## Conclusions

Recent years have seen a rapid development of theoretical models for the description of strong light–matter coupling and polaritonic chemistry in particular. Most theoretical work focuses on model systems, which is natural given the young age of the field, and yet this poses a major challenge as overly simplified models are unable to truly connect to experiment. Here, we illustrate a combination of *ab initio* trained ML models and modular cavity MD that aims to describe realistic molecules in realistic optical environments. This work allows, for the first time, the direct correlation of theoretical predictions to the experimentally measured changes in chemical kinetics.

We describe theoretically the appearance of single-molecule strong coupling and its influence on the Si–C bond for the experimentally investigated S_N_2 reaction.^14^ A clear frequency dependence of the rate constant, a critical aspect of polaritonic chemistry, is observed and translates into changes in enthalpy and entropy that are consistent with the experimental observations. Interestingly, we observe inhibiting and *catalyzing* effects for the same reaction, the latter of which stand in contrast to previous *ab initio* calculations.^13^ In total, three different regimes can be identified that are set apart by differences in the kinetic changes. (i) A strongly inhibiting effect without a clear vibrational contribution results in a strong increase in enthalpy but relatively small increase in entropy. (ii) Vibrationally supported catalysis predominantly increases the entropy and only slightly lowers the enthalpy, which results effectively in a simple shift in the Eyring plot, but we emphasize that the shorter reaction time likely results in an overestimation of this effect. (iii) Vibrationally supported inhibition raises the enthalpy and results in the strongest increase in entropy. The latter observation is qualitatively consistent with experiment and suggests a slight change in the chemical character of the reaction. Vibrationally supported rate changes (ii, iii) are accompanied by a more pronounced change in the normal-mode occupation in optically active domains, suggesting that the microscopic mechanism is caused by a stronger interplay of optically active modes via the cavity, i.e., by cavity mediated changes in the redistribution of vibrational energy.

The discrepancy with *ab initio* calculations, although sharing comparable patterns when scanning the cavity frequency and comparing identical ensembles, suggests that dynamic changes in electronic polarization induced by nuclear motion and mediated by the cavity play a considerable role at the selected coupling strength. This might explain why many simplified MD simulations aiming to understand vibrational strong coupling have been able to capture some frequency dependence but often showed strong detuning, or simultaneous catalyzing and inhibiting features for the same reaction, which, to the best of our knowledge, is in conflict with current experimental work. Our work demonstrates therefore the importance of *ab initio* QED in the future of polaritonic chemistry and emphasizes the significance of theory that is tailored to describe experimentally relevant reactions. Future development will focus on (self-consistent) perturbative corrections and the full transfer of the established framework to GPUs, thus providing access to realistic (optical) environments and an explicit description of solute–solvent ensembles. The latter is essential for investigating potential modification of solvation dynamics induced by strong coupling.^18,88,90^

Polaritonic chemistry remains an equally fascinating and puzzling domain of research. While major questions are yet to be answered,^2−4,6^ especially the connection between local chemistry and collective coupling as well as the interplay with solvation, the continuous growth of theoretical methodology and additional experiments draw an optimistic picture for the future of polaritonics. Our work adds to this a new facet and a clear perspective for possible future development, providing a valuable insight that can be experimentally validated.

## Data Availability

Training data, inputs, and final NEP models are available via Zenodo 10.5281/zenodo.10255268.

## Supporting Information Available

Information on the training procedure and performance estimates for the NEP models, numerical and simulation details, derivation and implementation of optical forces, *NVT* calculations with corresponding estimates for enthalpy and entropy, details for kinetic estimates from *NVE* calculations (including Arrhenius plots), details and results for rate and Si–C distance estimates for consistency check, details for normal-mode projections and Si–C stretching contributions, units, second evaluation of the consistency check based on a different NEP model trained from DFT calculations using the smaller 6-31G* basis, and extensive information and data on the calculation of vibrational frequencies (intermediate and transition states) (PDF)

## Footnotes

a Note in Figure 3 of ref (87) the ∝1/ω decay for fixed *V*_c_, i.e., with fixed *g*_0_/ω_c_, one would obtain *g*_0_^2^/ω_c_ = *g*_0_^2^/ω_c_^2^ω_c_ ∝ ω_c_.
