# Supplementary material for: Machine Learning for Polaritonic Chemistry: Accessing Chemical Kinetics
Source: J Am Chem Soc. 2024 Feb 14;146(8):5402–13. doi: 10.1021/jacs.3c12829 (PMC10910569; doi:10.1021/jacs.3c12829)
Supplement: Supplementary file 1 — ja3c12829_si_001.pdf [file ja3c12829_si_001.pdf]

Supplementary Information to Machine Learning for Polaritonic Chemistry: Accessing chemical kinetics

Christian Schäfer,[1, 2, *] Jakub Fojt,[1] Eric Lindgren,[1] and Paul Erhart[1]

[1] *Department of Physics, Chalmers University of Technology, 412 96 Göteborg, Sweden*
[2] *Department of Microtechnology and Nanoscience, MC2, Chalmers University of Technology, 412 96 Göteborg, Sweden*

(Dated: January 23, 2024)

I. SUPPLEMENTARY METHODS

A. Preparing the training set

We use an active learning approach in this work. An initial training set includes unrelaxed structures from the potential energy surface published in Ref. [1] and additional structures generated by rattling the minimum energy $PTAF^-$ structure. We then used the ORCA code version 5.0 [2] (PBE, 6-31G* basis) to obtain energies, forces, and dipoles. We trained a first version of the NEP model, using the NEP-3 potentials in GPUMD [3]. With this first generation model, we performed molecular dynamic simulations and selected trajectories with bad performance, adding those structures to the training set and repeating the procedure for a total of 7 generations. It should be noted that GPUMD has been undergoing changes since those initial attempts.

The comparably small 6-31G* basis provided reasonable energies but showed limited reliability for the dipole moments. We prepared a new dataset that included all rattling and molecular dynamics structures as well as a randomly sampled set of structured from the potential energy surface. We performed DFT PBE def2-TZVP calculations (using tight SCF convergence) to generate in total 20170 structures to train the final dipole NEP model with the following parameters.

```
mode      1
version   4
type      4 Si F C H
cutoff    8 6
n_max     15 8
neuron    80
batch     500000
generation  500000
```

The energy/force model has been trained on the same dataset with the input parameters.

```
version  4
type  4 Si F C H
cutoff  8 4
n_max  8 6
l_max  4 0
lambda_1  0.1
lambda_2  0.1
lambda_e  1
lambda_f  3
lambda_v  0
neuron  40
batch  300000
generation  500000
```

Both models are sufficiently converged and their performance is illustrated in the following sections.

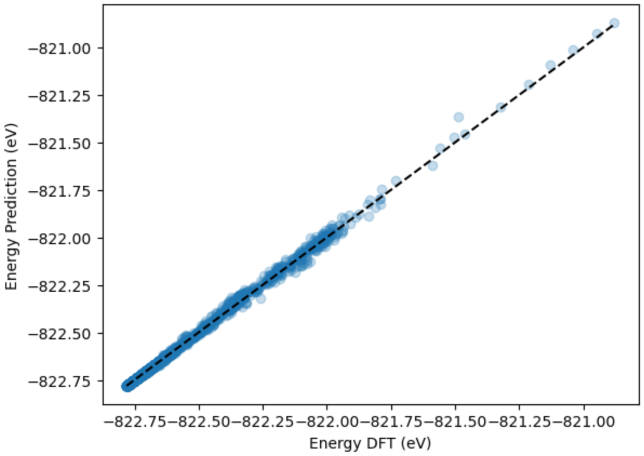

Supplementary Figure 1. Simple scatter-plot for the energy model. We use here all training structures but show additional validation checks in the following sections.

B. Initial performance estimates

Let us start our model evaluation with the simple scatter plot Fig. 1 for the expected and predicted energies. The model is well converged within its training set. Additional tests for an independent test-set follow later.

We compared our dipole model against the established symmetry adapted Gaussian Process Regression (SA-GPR) employed by TENSOAP [4] for which we randomly selected a set of 993 structures. Fig. 2 shows dipole predictions for GPUMD and TENSOAP. SA-GPR is considerably slower and practical application for molecular dynamics is limited as it requires at each step to build kernel elements between the test and training set. That

* Electronic address: christian.schaefer.physics@gmail.com

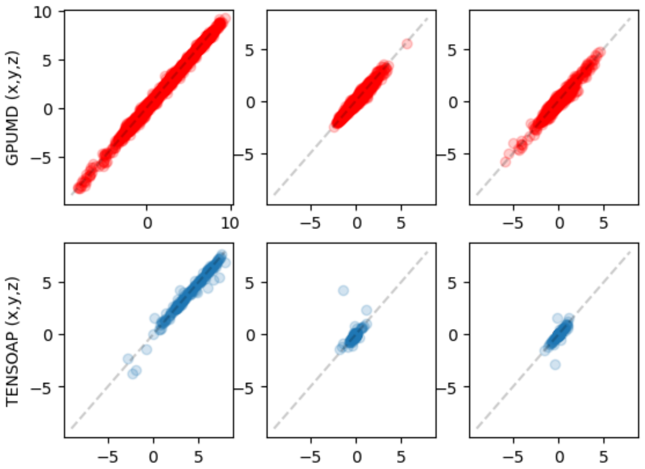

Supplementary Figure 2. Quality assessment of the prediction for the x,y, and z component (left to right) of the GPUMD and TENSOAP model. We use all 20170 structures for the GPUMD scatter plot. The SA-GPR model of TENSOAP used 800 structures for training and 193 for the here shown scatter plot.

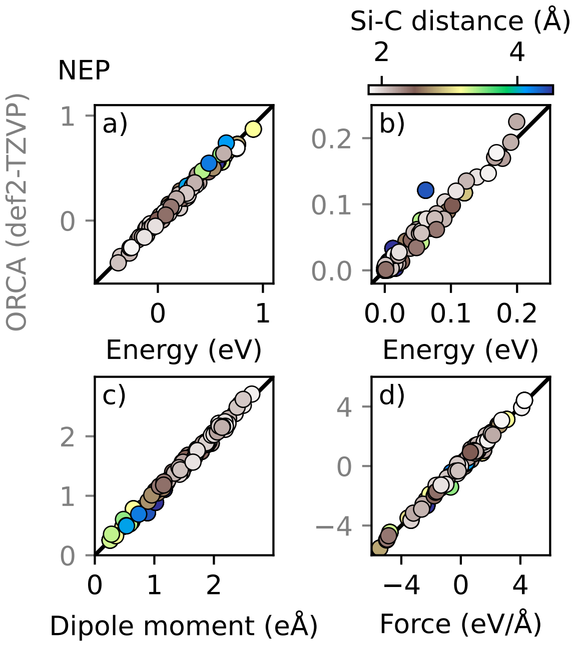

Supplementary Figure 3. Parity plots for our NEP model versus ab initio calculations performed with ORCA and the def2-TZVP basis set. Comparison of (a) potential energies of the electronic system (we have subtracted $-22\,214\,\mathrm{eV}$ from the values), (b) kinetic energies of the electronic system, (c) dipole moments and (d) the (electronic + cavity) force acting on the Si-C bond projected on the bond vector.

said, they require typically less data. For this reason, we decided to use less data for SA-GPR which keeps the training time and memory requirements low and allows for somewhat comparable evaluation times, would one perform molecular dynamics with both models.

Our NEP model performs overall well and is quicker to evaluate than SA-GPR, making it the more convenient choice for our purpose. This short discussion is not suited to make a general claim about the superiority of one of the models but merely serves as sanity check among the existing approaches.

C. Validation of the NEP models compared to ab initio calculations

To validate our NEP model against data outside the training set, we select 99 configurations for various trajectories, at various times, from our MD simulations performed in SI Sec. II D. This provides an estimate for the quality of the model in general and the specific reliability of our reruns from which we draw the conclusion that electronic polarization might play a more relevant role than currently thought. For each configuration we evaluate the potential and kinetic energies of the electronic system, the dipole, and the total force (both electronic and cavity contributions) on the Si-C bond using the NEP model, and using ORCA (Figure 3). NEP model and ORCA are overall in good agreement, and we can expect that our NEP model is able to accurately reproduce the trajectories that one would obtain from *ab initio* MD. There is one outlier among the data for the kinetic energy featuring a large Si-C bond distance (bond is broken). ML models are excellent at interpolation but configurations that push beyond the tightly sampled domain will suffer from reduced accuracy. However, those configurations do not play any role in our analysis since the bond is considered as broken once it crosses a Si–C distance of 3.5Å, i.e., any data generated for larger values do not enter into our analysis.

We perform additional consistency checks between the DFT codes ORCA and NWChem with the def2-TVZP basis set (Figure 4) and for two different basis sets def2-TVZP and 6-31G* within NWChem (Figure 5). ORCA and NWChem are in good agreement, besides a small and irrelevant constant shift in the potential energy. However, the difference between the basis sets is larger than the difference between NEP model and reference calculations, which provides further evidence for the quality of our model in the relevant domain.

D. Numerical Details

All ASE calculations use the Velocity Verlet propagator with a time-step of 0.1 fs. We obtained the Jacobian of the dipole moment with a 2nd-order central-difference approximation using displacements $h = 10^{-4}$ Å, implemented in the Python package calorine [5]. This value is located at the beginning of a stable plateau illustrated in Fig. 6, i.e., smaller steps are not useful.

The ensemble averaged energy loss during propaga-

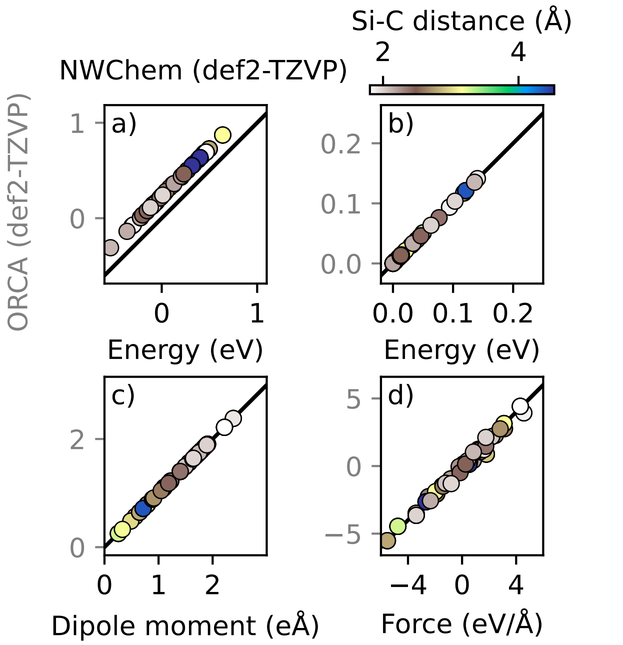

Supplementary Figure 4. Parity plots for ab initio calculations performed with ORCA versus NWChem. The def2-TVZP basis was used in both cases. Comparison of (a) potential energies of the electronic system (we have subtracted $-22\,214\,\mathrm{eV}$ from the values), (b) kinetic energies of the electronic system, (c) dipole moments and (d) the (electronic + cavity) force acting on the Si-C bond projected on the bond vector.

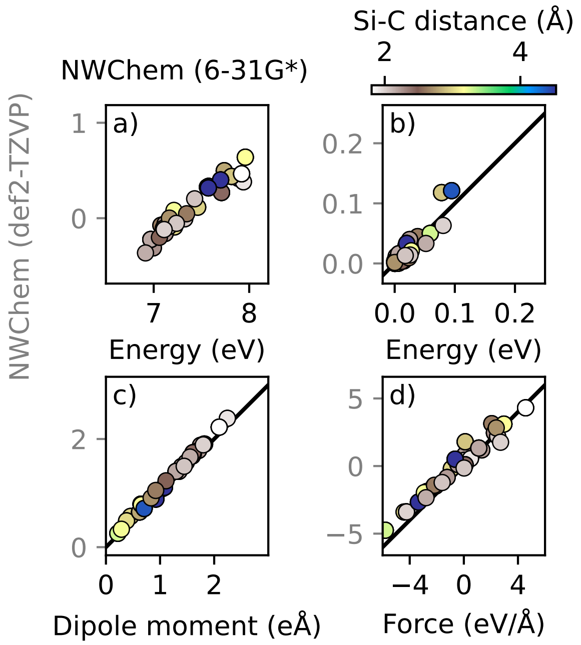

Supplementary Figure 5. Parity plots for ab initio calculations using the def2-TVZP versus 6-31G* basis sets. Calculations were performed using NWChem. Comparison of (a) potential energies of the electronic system (we have subtracted $-22\,214\,\mathrm{eV}$ from the values), (b) kinetic energies of the electronic system, (c) dipole moments and (d) the (electronic + cavity) force acting on the Si-C bond projected on the bond vector.

tion is given in Fig. 7. It increases with increasing frequency since the ratio g/ω is kept constant and a larger cavity frequency leads thus to stronger cavity induced forces which in turn increase the accumulated error due to the finite-difference approximation. We plan to extend GPUMD with analytic derivatives in the future which would entirely mitigate this error.

Our NVE calculations set a temperature by sampling initial velocities from a Boltzmann distribution and removing the center of mass momentum. Ref. [1] showed that considering a solvent resulted in notable changes of the relative infrared activity of vibrational excitations but the good agreement in enthalpy (using NVT conditions) suggests that the effect on the reaction is small enough to draw relevant conclusions from our investigations.

II. SUPPLEMENTARY INFORMATION

A. Obtaining total forces from electronic forces and dipoles

Splitting the Hamiltonian in matter $\hat{H}_0$ and light-matter component

$$\hat{H} = \hat{H}_0 + \frac{1}{2}[\hat{p}^2 + \omega_c^2(\hat{q} - \frac{1}{\sqrt{\varepsilon_0 V_c}}\varepsilon_c \cdot \hat{\boldsymbol{\mu}}/\omega_c)^2] \quad (1)$$

and introducing the simplified classical limit $\hat{q}, \hat{p}, \hat{\boldsymbol{\mu}} \rightarrow q, p, \boldsymbol{\mu}$, we obtain the classical Hamilton function

$$\mathcal{H}_{LM} = \frac{1}{2}[p^2 + \omega_c^2(q - \frac{1}{\sqrt{\varepsilon_0 V_c}}\varepsilon_c \cdot \boldsymbol{\mu}/\omega_c)^2]. \quad (2)$$

The careful reader will notice that the classical nuclear limit does not strictly imply $\hat{\boldsymbol{\mu}} \rightarrow \boldsymbol{\mu}$ since the total dipole moment includes electronic and nuclear contributions. The electronic remainder, especially $(\varepsilon_c \cdot \hat{\boldsymbol{\mu}}_e)^2 + \varepsilon_c \cdot \hat{\boldsymbol{\mu}}_e \varepsilon_c \cdot \boldsymbol{\mu}_n$, polarizes the electronic system and thus influences the nuclear forces. We discuss the potential consequences of this subtlety and the formally correct treatment in terms of the cavity Born-Oppenheimer [6] in further detail in the main text.

Following classical Hamilton mechanics for the canonical displacement mode of the cavity oscillator

$$\partial_t p = \{p, \mathcal{H}_{LM}\} = -\frac{\partial \mathcal{H}}{\partial q} \quad (3)$$

$$= -\omega_c^2 q + \omega_c \frac{1}{\sqrt{\varepsilon_0 V_c}}\varepsilon_c \cdot \boldsymbol{\mu} \quad (4)$$

provides, due to the equivalence of kinetic and canonical momentum in Power-Zienau-Wooley gauge $\partial_t q = \{q, \mathcal{H}_{LM}\} = \frac{\partial \mathcal{H}}{\partial p} = p$, the mode-resolved Maxwell equation

$$(\partial_t^2 + \omega_c^2)q(t) = \omega_c \frac{1}{\sqrt{\varepsilon_0 V_c}}\varepsilon_c \cdot \boldsymbol{\mu}(t), \quad (5)$$

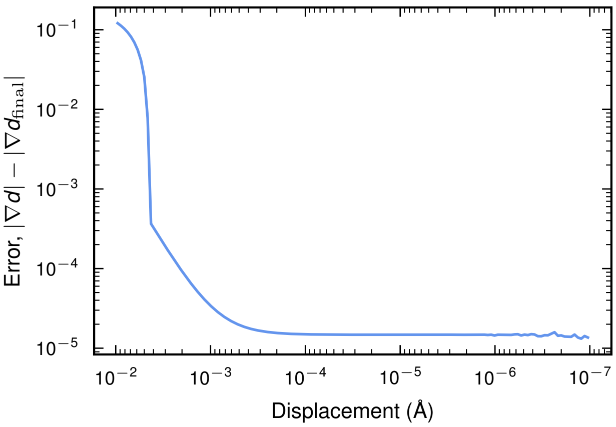

Supplementary Figure 6. Finite-difference error as a function of finite difference displacement size. The error is calculated as the difference between the norm of the dipole gradient for a certain displacement and for the smallest displacement 10^{-8} Å. Note that the error plateaus for displacements smaller than 10^{-4} Å, which could be an effect of reaching the limit of the accuracy for the dipole predictions from the model.

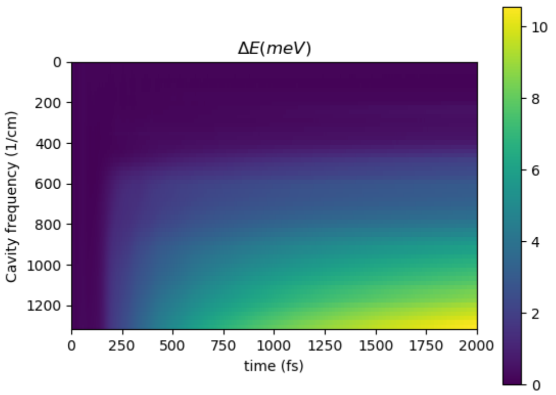

Supplementary Figure 7. Average energy loss in trajectory bundle over time for 400 K and $g/\omega = 1.132$.

solved by Green's function $G(t-t') = \frac{\sin(\omega_c(t-t'))}{\omega_c}$.

Enforcing zero initial cavity momentum $p(0) = 0$, the cavity mode displacement $q(t)$ depends then on the time-evolution of the molecular dipole moment through

$$q(t) = q(0)\cos(\omega_c t) + \int_0^t \frac{\varepsilon_c \cdot \boldsymbol{\mu}(t')}{\sqrt{\varepsilon_0 V_c}} \sin(\omega_c(t-t'))\,\mathrm{d}t'. \quad (6)$$

The initial mode displacement is chosen such that the initial optical force is zero, i.e., $q_0 = \frac{1}{\omega_c}\frac{1}{\sqrt{\varepsilon_0 V_c}}\varepsilon_c \cdot \boldsymbol{\mu}_0$. To avoid having to store the entire time-evolution of $\boldsymbol{\mu}$ during MD, we decouple t and t' in Equation 6

$$q(t) = q(0)\cos(\omega_c t) + \sin(\omega_c t)C(t) - \cos(\omega_c t)S(t) \quad (7)$$

$$C(t) = \int_0^t \frac{\varepsilon_c \cdot \boldsymbol{\mu}(t')}{\sqrt{\varepsilon_0 V_c}} \cos(\omega_c t')\,\mathrm{d}t' \quad (8)$$

$$S(t) = \int_0^t \frac{\varepsilon_c \cdot \boldsymbol{\mu}(t')}{\sqrt{\varepsilon_0 V_c}} \sin(\omega_c t')\,\mathrm{d}t', \quad (9)$$

and update the integrals during MD as

$$C(t+\Delta t) = C(t) + \int_t^{t+\Delta t} \frac{\varepsilon_c \cdot \boldsymbol{\mu}(t')}{\sqrt{\varepsilon_0 V_c}} \cos(\omega_c t')\,\mathrm{d}t' \quad (10)$$

$$S(t+\Delta t) = S(t) + \int_t^{t+\Delta t} \frac{\varepsilon_c \cdot \boldsymbol{\mu}(t')}{\sqrt{\varepsilon_0 V_c}} \sin(\omega_c t')\,\mathrm{d}t', \quad (11)$$

where the trapezoidal rule is used to approximate the integrals.

We emphasise that $\boldsymbol{\mu}$ and $\boldsymbol{F}_{\rm PES}$ can be obtained either directly from DFT, or our NEP models, but only the latter is computationally tractable for MD simulations.

B. NVT reference calculation

Theoretically predicted rates require sufficient time for thermalization to reach a statistically meaningful distribution near an equilibrium state of the system. This requires long propagation times and suggests the use of NVT conditions. Both aspects are problematic in calculations involving the cavity for two major reasons. First, the interplay between thermostat and cavity might give rise to spurious features that misguide our interpretation. Second, observing an appreciable number of reactions under such conditions requires long propagation times. The latter is not an issue for calculations on the GPU, but the current CPU based cavity calculator is certainly limited in this aspect.

Fig. 8 presents an example of the number of reactant molecules over time at 400K. We observe two characteristic domains, an initial burst of reactions, and a second domain for the properly thermalized reactant. We extract the rates as linear fits to the latter and calculate a transition-state enthalpy of $\Delta H^{\ddagger} = 0.345$ eV from the Eyring plot in Fig. 9, which is in agreement with experimental estimates $\Delta H^{\ddagger} = 35 \pm 4$ kJ/mol. [7] It should be noted that we only estimate the Si-C breaking reaction step here, as the correct fluoride attacking and final protonation steps would require an explicit treatment of the solvent.

C. Rate calculations and thermodynamics from NVE

A reaction event took place when the Si–C bond is stretched beyond a value of 3.5 Å. This is located about 0.5 Å behind the transition state, allowing us to account

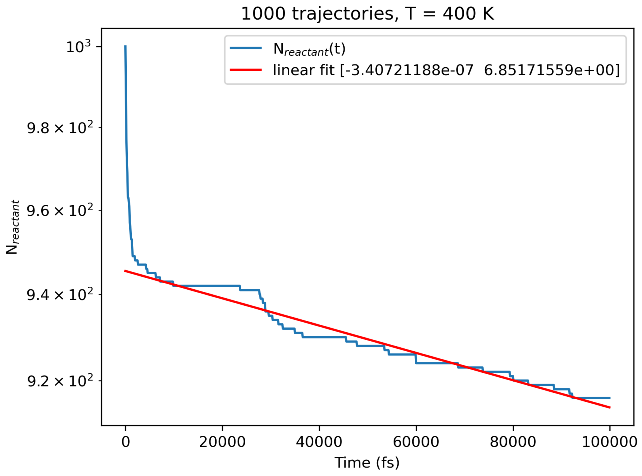

Supplementary Figure 8. Log-plot of the number of reactant molecules $PTAF^-$ vs time and linear fit to the domain after initial equilibration. NVT conditions with 400K were enforces using the GPUMD internal Nosé-Hoover chain thermostat with relaxation time-value of 100. A time-step of 0.1 fs was used.

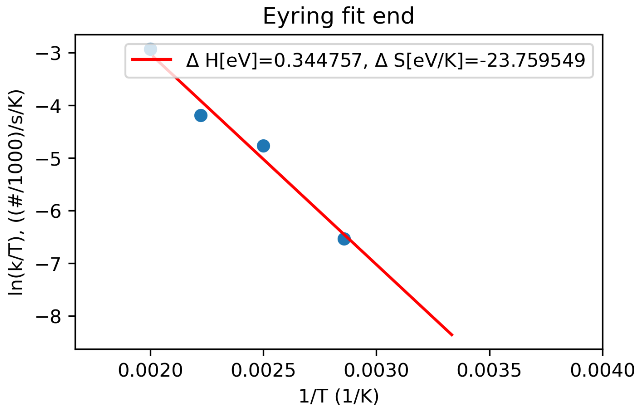

Supplementary Figure 9. Eyring plot and fit for the unidirectional reaction $PTAF^- \rightarrow FtMeSi + PA^-$ obtained under NVT conditions according to Fig. 8. Fit to the thermalized reaction dynamics. The latter predicts an enthalpic barrier of $\Delta H^\ddagger = 0.345$ eV, which is consistent with the experimentally measured $\Delta H^\ddagger = 35 \pm 4$ kJ/mol. [7]

for recrossing events in a simplified manner. Once a trajectory showed such a reaction event, it is considered as product in the following. The rate is calculated as number of products after 2 ps. Our rate constant is therefore that of a unidirectional reaction towards the product and does not correspond to the equilibrium rate. Rate constant is calculated for different temperatures, recall that the initial velocities are samples from the Maxwell-Boltzmann distribution, and plotted in the Eyring plots. The linear fits with a first-order polynomial provides estimates for enthalpy and entropy for this reaction process. The thermodynamic quantities are presented as absolute difference to the cavity-free calculations.

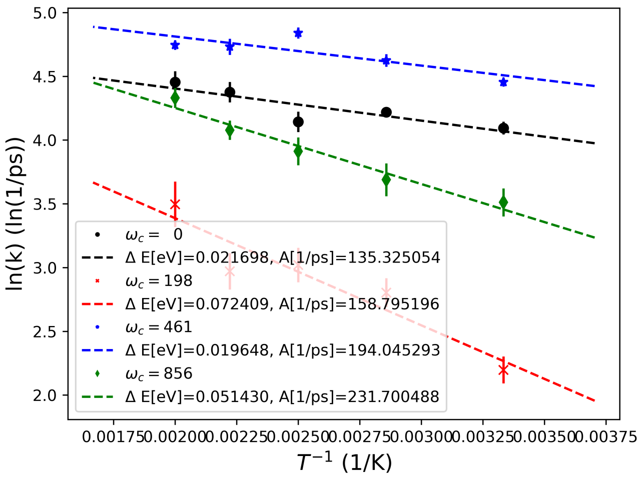

Supplementary Figure 10. Arrhenius plot $k = Ae^{-\Delta E/k_BT}$ for the unidirectional reaction $PTAF^- \rightarrow FtMeSi + PA^-$ obtained under NVE conditions.

We point out that our initial state is energetically above the transition-state, i.e., the reaction is almost barrier-free. This results in the positive enthalpy shown in the Eyring plot (main document) and the low activation barrier in the Arrhenius plot Figure 10. We plan to provide a rigorous NVT equilibrium rate once a full GPU implementation is available.

D. Consistency Checks

Starting the same initial conditions as Ref. [1], we have calculated rates and average Si–C distances. The obtained rates (Fig. 11, top) are largely consistent with our observations in this work and are further discussed in the main text. Fig. 11 middle shows the Si–C distance averaged over the full ensemble and the specified time-domain. Fig. 11 bottom shows the same but only for the subset of trajectories that are reactive outside the cavity. The *ab initio* calculations in Ref. [1] used a short integration domain of 0.7 ps and utilized only the subset of trajectories reactive outside the cavity, i.e., the most consistent comparison is with the blue solid line in Fig. 11 bottom. Ref. [1] did not indicate any rate enhancing effect of the cavity, rate and Si–C distance are sufficiently correlated to draw a connection.

We re-optimized the TS and calculated the vibrational modes at the transition state. The frequency corresponding to the negative TS curvature is with 73.38 cm^{-1} close to previously reported values [1, 8, 9]. In order to check if our NEP model informs the molecular dynamics simulations with the correct value, we fixed the Si-C atoms and performed a short BFGS optimization with our NEP model starting from the TS structure obtained in ORCA. The calculated value of 69.5 cm^{-1} (see SI Sec. III) is in close agreement and suggests that any effect originating

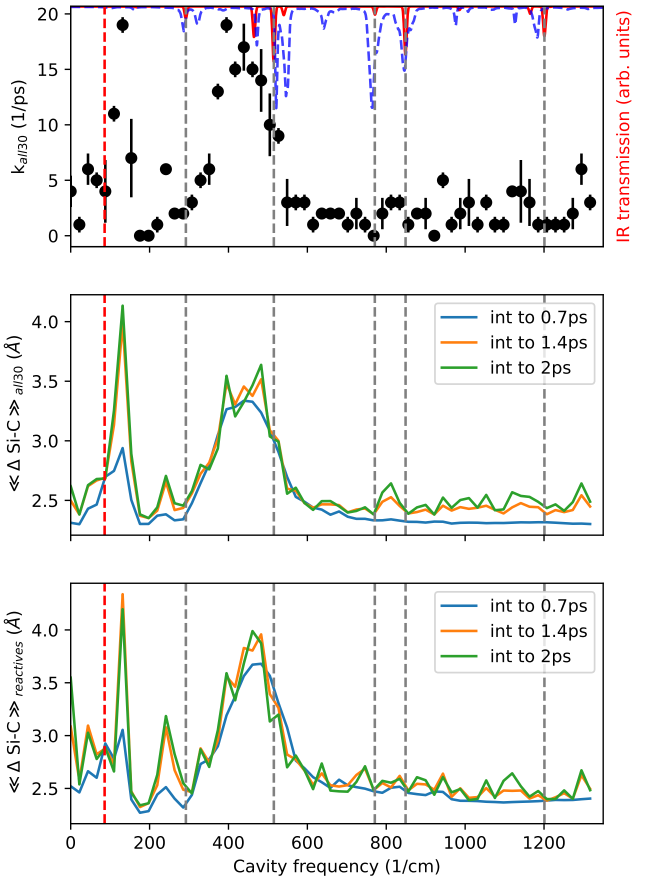

Supplementary Figure 11. Top: Rate for the unidirectional reaction $PTAF^- \rightarrow FtMeSi + PA^-$ using the 30 initial configurations used in Ref. [1] and $g/\omega = 1.132$, but propagated with our NEP based molecular dynamics calculator. Transmission spectrum obtained from Octopus at 0K, harmonic approximation (red solid), and using our NEP model and GPUMD at 400K NVE conditions (blue dashed). Vertical lines indicate characteristic features observed in Ref. [1].
Middle: Trajectory and time-averaged Si-C distance using all 30 trajectories for different time-intervals.
Bottom: Trajectory and time-averaged Si-C distance for the subset of reactive trajectories for different time-intervals. This corresponds to the observable shown in Ref. [1]. The relatively clear correlation with the rate supports the reliability of the averaged Si-C distance as indicator for rate changes. A sanity check for the difference between middle and bottom picture is, that the average Si–C distance at $\omega_c = 0$ (outside cavity) is clearly higher for the subset of reactive trajectories which increases their average value. The overall differences are small, rate and Si–C distance show a strong correlation.

from the transition-state curvature should be correctly accounted for. Future work should investigate how the cavity Born-Oppenheimer approximation modifies our observation, as we would expect then a closer agreement with previous model and *ab initio* calculations [1, 10].

E. Normal mode occupations

Fig. 12 illustrates the difference in normal mode occupation for 3 different choices of cavity frequency. The normal mode occupation $\mathbf{o}$ is calculated by normalizing the force extracted from the trajectory at a given time $\mathbf{f}(t) = \mathbf{F}(t)/\|\mathbf{F}(t)\|_2$ and projecting it onto the orthornormal set of normal mode forces $\mathbf{o} = (\mathbf{f}_{nm} \cdot \mathbf{f}(t))^2$, $\mathbf{f}_{nm}^T \cdot \mathbf{f}_{nm} = \mathbb{1}$. Notice that the y-axis uses the frequency and we stretch the normal-mode occupation accordingly. The overall structure for $\omega_c = 856$ cm^{-1} is comparable to Ref. [1]. For comparison, one should take into account, that the experimentally most relevant normal mode is located at 849 cm^{-1} in Ref. [1] while it is located at 831 cm^{-1} when using our NEP potential and the ASE internal vibrational mode calculator (0 K). Noticeable is the rather unaffected region around 750-1000 cm^{-1} from which only the optically active modes at 770 and 831 cm^{-1} stand out. The domain between 450-550 cm^{-1} is quite pronounced (similar to Ref. [1]).

F. Si–C stretching contribution in vibrational modes

Since the reactive step involves breaking the Si–C bond, the contribution of Si–C stretchings in the normal modes is an important indicator for the expected impact on the reaction when energy is redistributed between optically active modes. Figure 13 demonstrates that, in agreement with Fig. 2C from the main manuscript, the Si–C stretching contributions are located foremost in the energy-window between 160 cm^{-1} and 840 cm^{-1}, with the exception of the C=C bond stretching around 1200 cm^{-1} and a high-energy mode beyond above 2000 cm^{-1}. Nonetheless, this supports our argumentation that the reactive modes that could be effected of dynamic electronic polarization are localized below 840 cm^{-1} which explains why changes at higher frequencies disappear.

G. Unit conversion between atomic units and ASE units

The dimensionless ratio g_0/ω_c determines our coupling strength. It should be noted that using the implemented cavity-calculator requires the coupling $\lambda = \frac{1}{\sqrt{\varepsilon_0 V_c}}$ in ASE units. Our scripts handle this conversion automatically but we provide a brief discussion of the relevant conversion to facilitate reproduction from independent researchers. Since $g_0/\omega_c = [\mu]\sqrt{1/\hbar\omega_c 2\varepsilon_0 V_c}$, where $[\mu]$ denotes the units of the dipole moment, and thus $[g_0/\omega_c] = [1] = [\mu][\lambda]/\sqrt{[\hbar\omega_c]}$ as well as $[\lambda] = \sqrt{[\hbar\omega_c]}/[\mu]$. Furthermore, $\lambda^{au} = \frac{g_0^{au}}{\omega_c^{au}}\sqrt{2\omega_c^{au}}$ and we finally arrive at $\lambda^{ase} = \frac{g_0^{au}}{\omega_c^{au}}\sqrt{2\omega_c^{au}}\frac{\sqrt{[energy]_{au\ to\ ase}}}{[length]_{au\ to\ ase}}$ ($[x]_{au\ to\ ase}$ are con-

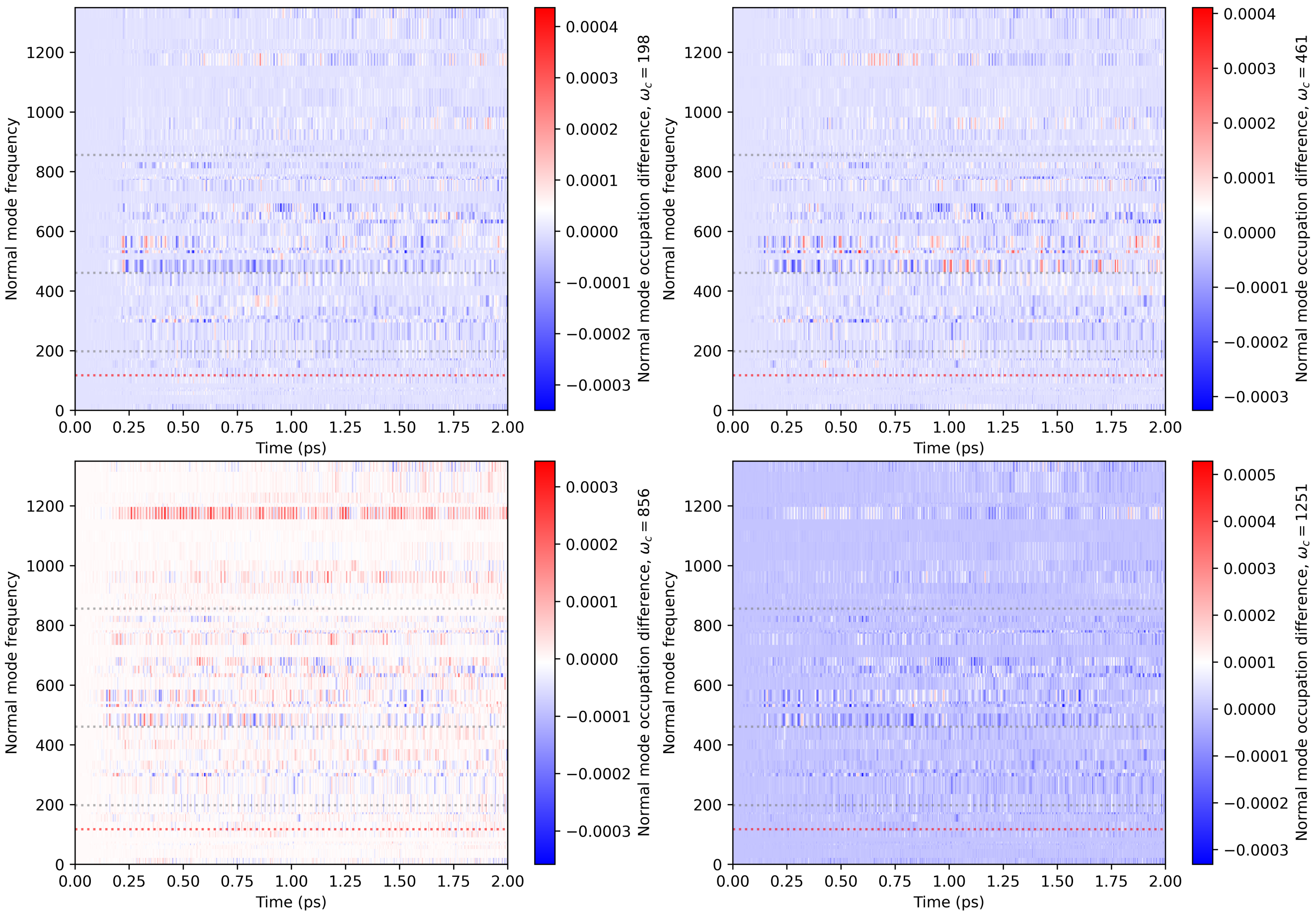

Supplementary Figure 12. Top left: Difference in normal mode occupation for $\omega_c = 198$ cm^{-1} and $g/\omega = 1.132$ vs free space at 400 K. Top right: Difference in normal mode occupation for $\omega_c = 461$ cm^{-1} and $g/\omega = 1.132$ vs free space at 400 K.
Bottom left: Difference in normal mode occupation for $\omega_c = 856$ cm^{-1} and $g/\omega = 1.132$ vs free space at 400 K. Bottom right: Difference in normal mode occupation for $\omega_c = 1251$ cm^{-1} and $g/\omega = 1.132$ vs free space at 400 K.

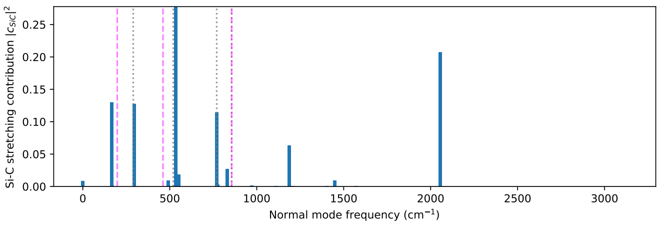

Supplementary Figure 13. Si–C contribution to vibrational normal modes. Obtained by projecting a force $\mathbf{f}_{SiC} = -\frac{1}{\sqrt{2}}\mathbf{e}_x^{Si} + \frac{1}{\sqrt{2}}\mathbf{e}_x^{C}$ on the normal-mode forces (equivalent to Figure 12).

versions between units) which can be directly related to Ref. [1] by taking $\frac{g_0^{au}}{\omega_c^{au}} = 1.132$.

H. NEP model using a smaller electronic basis

As detailed in Sec. I A, we started training a second NEP model based on DFT calculations (using ORCA) with the smaller 6-31G* basis set. Dipole moments and stretched configurations are less reliable when using the small 6-31G* basis, such that the following presentation should be consider with caution. As shown in Figure 14, the trained NEP models based on the 6-31G* calculations accurately reproduce the forces obtained from ORCA.

The smaller basis results in a lower reaction barrier and thus a quick saturation of the limited set of trajectories. Nonetheless, we can use those NEP models to investigate if the dynamic electronic polarization remains an important components resulting in comparable deviations.

Figure 15 presents reaction rate constants and Si–C distances obtained from starting ML+MD calculation using the 6-31G* trained NEP models. Two aspects require additional discussion:

1. The overall shape is consistent with Figure 11 for the average change in Si–C distance in the first 700 fs (blue lines in middle and bottom plot). The catalysing character around 450/cm is smaller and seems to be broader, such that the domain around 800/cm obtains additional weight, better emphasizing the resonant dependence. The overall effect remains small at larger frequencies.

2. All frequencies besides 200/cm quickly reach comparable number of products as obtained at the catalysing frequencies explained in the first aspect, i.e., the lower barrier allows the non-catalyzed trajectories to catch up. The only feature that is clearly standing out after 2 ps is the strong inhibition at 200/cm (in Si–C averages and rate).

The basic qualitative behavior of both ML+MD investigations is consistent in the first 700 fs. However, the quick increase at all frequencies (besides 200/cm) implies that only few features remain to play a role after longer propagation time or broader sampling, i.e., only the features

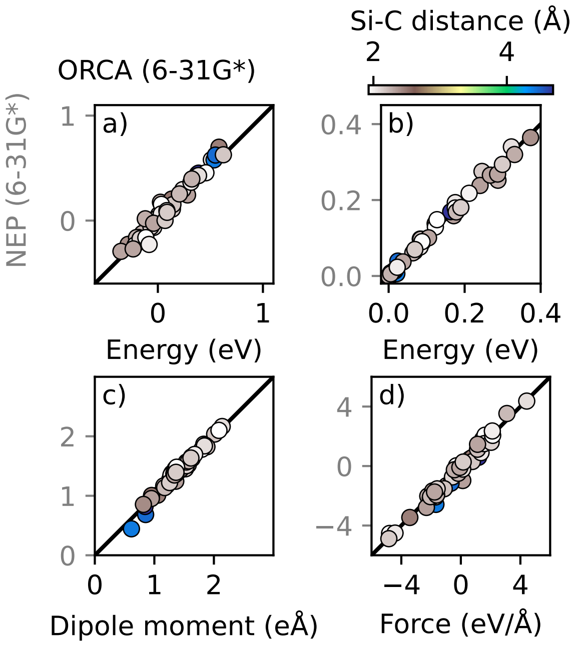

Supplementary Figure 14. Parity plots for the alternative NEP model trained on the 6-31G* basis set versus ab initio calculations performed with ORCA and the 6-31G* basis set. Comparison of (a) potential energies of the electronic system (we have subtracted $-22\,206.5\,\mathrm{eV}$ from the values), (b) kinetic energies of the electronic system, (c) dipole moments and (d) the (electronic + cavity) force acting on the Si-C bond projected on the bond vector.

reported in Fig. 2C of the paper can be expected to remain relevant after proper sampling. Nonetheless, that the qualitative trend of both ML+MD investigations (up to 700 fs) is consistent and qualitatively contradicts the QEDFT calculations (in the same 700 fs domain) supports our conclusions in the main paper.

I. Vibrational frequencies

Figure 16 presents the normal-mode frequencies and their difference obtained at the intermediate state (blue) and the transition-state (orange) when using our NEP model or ORCA (both based on def2-TZVP basis). The modes are sorted by their frequency.

The ORCA transition-state (TS) geometry was obtained by reoptimizing an initial gues using the "OptTS" flag. The hessian was calculated at the beginning of the optimization to ensure reliable convergence. Using our NEP model, the TS and its frequencies have been calculated by using the final TS structures from ORCA, fixing the positions of the two relevant Si-C atoms, performing a BFGS optimization to reduce forces down to a maximum value of 10^{-12}, and calculating the vibrational modes of this relaxed TS structure. The lowest TS frequency of NEP model and ORCA calculation are with

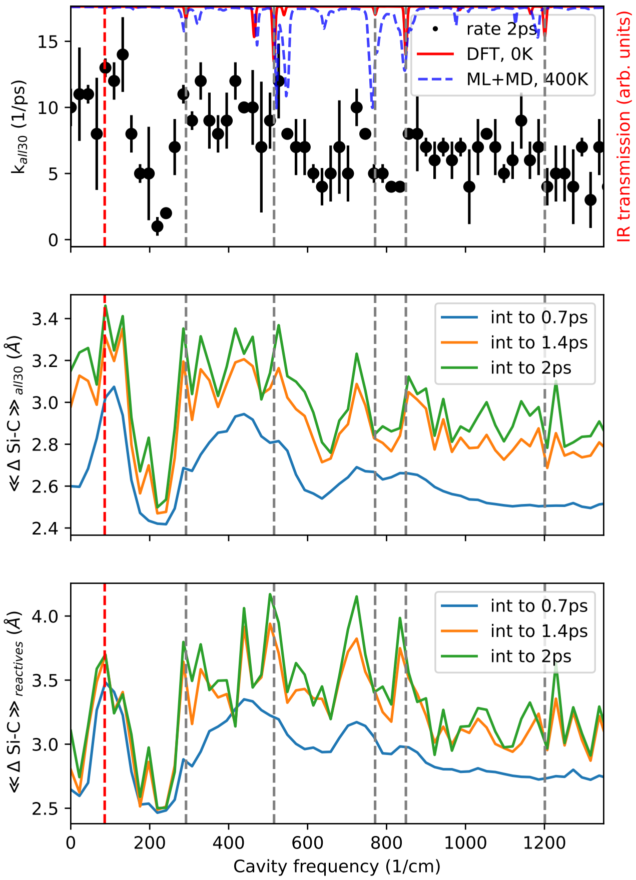

Supplementary Figure 15. Rate and Si–C averages corresponding to Figure 11 but obtained using the 6-31G* trained NEP models. Top: Rate for the unidirectional reaction $PTAF^{-} \rightarrow FtMeSi + PA^{-}$ using the 30 initial configurations used in Ref. [1] and $g/\omega = 1.132$, but propagated with our NEP based molecular dynamics calculator. Transmission spectrum obtained from Octopus at 0K, harmonic approximation (red solid), and using our NEP model and GPUMD at 400K NVE conditions (blue dashed). Vertical lines indicate characteristic features observed in Ref. [1].
Middle: Trajectory and time-averaged Si-C distance using all 30 trajectories for different time-intervals.
Bottom: Trajectory and time-averaged Si-C distance for the subset of trajectories used in Figure 11.

69.5 and 73.38 cm^{-1} in close agreement.

In addition, we provide below the explicit normal-mode frequencies.

a. Intermediate state vibrational frequencies NEP model:

#	meV	cm^-1
0	1.0i	7.8i
1	0.4i	3.1i
2	0.2i	1.9i
3	0.1i	0.5i
4	0.0i	0.1i
5	0.0i	0.0i
6	0.2	1.4
7	5.3	42.6
8	7.6	61.0
9	8.4	67.5
10	9.0	72.9
11	9.9	79.9
12	12.5	101.1
13	15.0	121.0
14	20.5	165.1
15	21.1	170.3
16	22.1	178.4
17	36.2	292.4
18	36.8	296.8
19	39.1	315.3
20	39.8	321.4
21	46.4	374.1
22	49.2	397.1
23	54.1	436.2
24	60.8	490.1
25	64.4	519.7
26	66.3	534.7
27	66.9	539.3
28	68.4	552.0
29	76.5	617.3
30	79.0	636.8
31	79.7	642.5
32	85.4	688.6
33	86.6	698.7
34	95.5	770.5
35	96.1	775.1
36	96.3	776.5
37	98.1	791.2
38	98.1	791.4
39	103.0	831.1
40	103.3	833.4
41	106.1	855.6
42	108.6	875.9
43	111.4	898.8
44	113.3	913.8
45	120.4	971.3
46	123.0	991.8
47	129.6	1045.1
48	138.0	1112.9
49	139.3	1123.2
50	147.2	1187.1
51	149.6	1206.3
52	149.9	1209.2
53	150.3	1212.1
54	158.4	1277.3
55	167.5	1351.3
56	172.6	1392.3
57	172.8	1393.7
58	174.1	1404.0
59	175.2	1413.3
60	175.6	1416.6
61	176.0	1419.4
62	176.1	1420.6

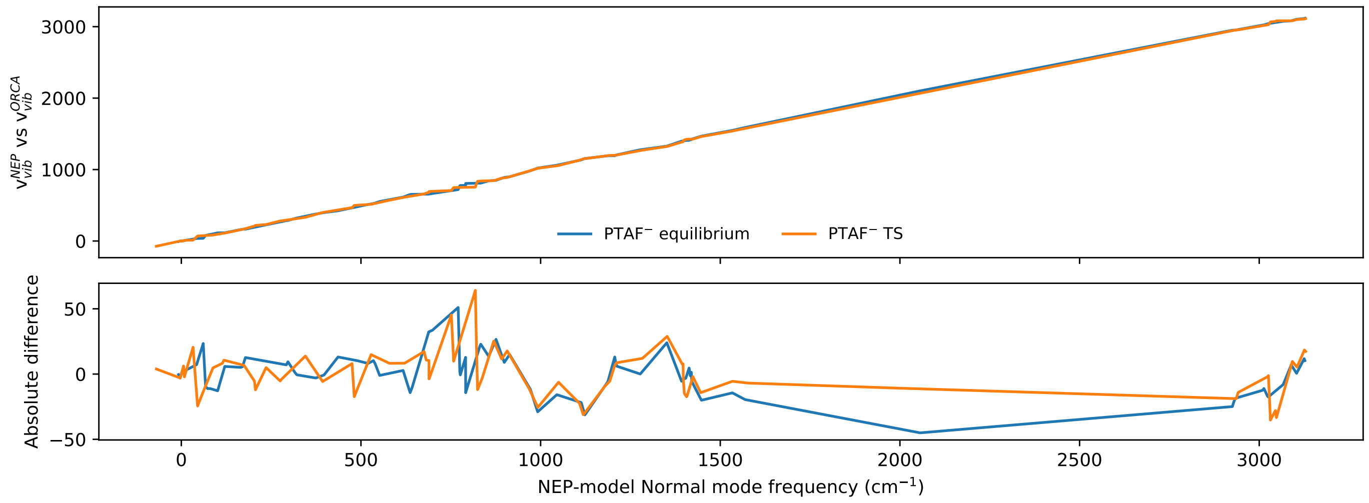

Supplementary Figure 16. Normal-mode frequencies and their difference obtained at the intermediate state (blue) and the transition-state (orange) when using our NEP model or ORCA (both based on def2-TZVP basis).

```
63  179.5    1447.5
64  190.2    1533.7
65  194.6    1569.2
66  254.9    2056.3
67  362.7    2925.3
68  363.2    2929.3
69  363.7    2933.2
70  373.2    3009.7
71  373.6    3013.1
72  373.6    3013.3
73  374.8    3023.2
74  375.0    3024.2
75  375.1    3025.2
76  380.2    3066.6
77  383.0    3089.1
78  384.8    3103.9
79  387.5    3125.2
80  387.7    3127.1
```

b. Intermediate state vibrational frequencies ORCA:

```
-----------------------
VIBRATIONAL FREQUENCIES
-----------------------
   0:         0.00 cm**-1
   1:         0.00 cm**-1
   2:         0.00 cm**-1
   3:         0.00 cm**-1
   4:         0.00 cm**-1
   5:         0.00 cm**-1
   6:        -7.42 cm**-1 ***imaginary mode***
   7:        35.37 cm**-1
   8:        37.55 cm**-1
   9:        77.07 cm**-1
  10:        84.03 cm**-1
  11:        90.97 cm**-1
  12:       113.94 cm**-1
  13:       115.14 cm**-1
  14:       159.98 cm**-1
  15:       164.68 cm**-1
  16:       165.73 cm**-1
  17:       285.36 cm**-1
  18:       287.37 cm**-1
  19:       313.75 cm**-1
  20:       321.97 cm**-1
  21:       377.11 cm**-1
  22:       397.83 cm**-1
  23:       423.11 cm**-1
  24:       479.85 cm**-1
  25:       511.57 cm**-1
  26:       524.41 cm**-1
  27:       531.28 cm**-1
  28:       553.05 cm**-1
  29:       614.53 cm**-1
  30:       651.06 cm**-1
  31:       652.71 cm**-1
  32:       656.27 cm**-1
  33:       665.17 cm**-1
  34:       719.49 cm**-1
  35:       773.21 cm**-1
  36:       777.17 cm**-1
  37:       778.38 cm**-1
  38:       805.68 cm**-1
  39:       809.53 cm**-1
  40:       810.52 cm**-1
  41:       842.00 cm**-1
  42:       849.28 cm**-1
  43:       889.89 cm**-1
  44:       898.81 cm**-1
  45:       982.89 cm**-1
  46:      1020.73 cm**-1
  47:      1060.90 cm**-1
  48:      1134.72 cm**-1
  49:      1154.47 cm**-1
  50:      1192.98 cm**-1
  51:      1193.18 cm**-1
  52:      1200.73 cm**-1
  53:      1206.08 cm**-1
  54:      1277.25 cm**-1
  55:      1327.29 cm**-1
  56:      1397.82 cm**-1
  57:      1398.03 cm**-1
  58:      1407.21 cm**-1
  59:      1408.67 cm**-1
  60:      1417.89 cm**-1
  61:      1417.98 cm**-1
  62:      1426.23 cm**-1
  63:      1467.57 cm**-1
  64:      1548.10 cm**-1
  65:      1588.76 cm**-1
  66:      2101.33 cm**-1
  67:      2950.21 cm**-1
  68:      2950.59 cm**-1
  69:      2951.78 cm**-1
  70:      3022.11 cm**-1
  71:      3024.15 cm**-1
  72:      3024.48 cm**-1
  73:      3040.26 cm**-1
  74:      3040.60 cm**-1
  75:      3042.77 cm**-1
  76:      3074.66 cm**-1
  77:      3082.10 cm**-1
  78:      3103.34 cm**-1
  79:      3113.39 cm**-1
  80:      3116.81 cm**-1
```

c. Transition state vibrational frequencies NEP model:

```
---------------------
  #    meV     cm^-1
---------------------
  0    8.6i     69.5i
  1    0.4i      3.0i
  2    0.0i      0.1i
  3    0.0i      0.1i
  4    0.1       0.5
  5    0.7       5.6
```

6	0.8	6.2
7	1.1	8.5
8	4.0	32.7
9	4.2	33.9
10	5.6	45.3
11	7.6	61.5
12	10.9	88.1
13	14.2	114.4
14	14.7	118.5
15	20.8	167.9
16	21.7	175.1
17	25.2	203.6
18	25.6	206.3
19	29.2	235.9
20	34.1	275.2
21	34.3	276.9
22	42.8	345.4
23	48.8	393.3
24	58.9	475.2
25	59.7	481.3
26	65.5	528.2
27	71.8	579.3
28	77.1	621.7
29	83.8	675.5
30	84.5	681.7
31	85.4	689.2
32	85.5	689.4
33	86.1	694.6
34	93.2	751.6
35	94.0	757.9
36	95.2	768.0
37	101.5	818.4
38	101.8	821.4
39	102.2	824.5
40	103.8	837.4
41	104.8	845.3
42	107.8	869.6
43	110.5	891.6
44	112.4	907.0
45	120.1	968.5
46	123.0	992.3
47	130.2	1049.9
48	137.4	1108.2
49	138.7	1118.8
50	146.2	1179.5
51	147.8	1192.4
52	149.4	1205.3
53	150.1	1211.0
54	159.1	1283.3
55	167.7	1352.3
56	173.0	1395.0
57	173.1	1396.5
58	173.2	1397.2
59	173.6	1400.0
60	174.3	1406.2
61	174.5	1407.6
62	176.5	1423.8
63	179.2	1445.5
64	190.2	1533.9
65	195.5	1577.1
66	248.6	2005.5
67	364.0	2936.1
68	364.6	2940.6
69	364.7	2941.6
70	374.8	3022.6
71	375.0	3024.9
72	375.1	3025.6
73	375.8	3031.2
74	377.5	3045.0
75	377.9	3047.8
76	380.4	3068.3
77	383.4	3092.3
78	385.0	3105.2
79	387.5	3125.7
80	388.0	3129.3

d. Transition state vibrational frequencies ORCA:

```
-----------------------
VIBRATIONAL FREQUENCIES
-----------------------
   0:         0.00 cm**-1
   1:         0.00 cm**-1
   2:         0.00 cm**-1
   3:         0.00 cm**-1
   4:         0.00 cm**-1
   5:         0.00 cm**-1
   6:       -73.38 cm**-1 ***imaginary mode***
   7:        10.65 cm**-1
   8:        12.17 cm**-1
   9:        17.68 cm**-1
  10:        69.77 cm**-1
  11:        74.63 cm**-1
  12:        83.36 cm**-1
  13:       106.14 cm**-1
  14:       107.85 cm**-1
  15:       160.73 cm**-1
  16:       168.58 cm**-1
  17:       209.02 cm**-1
  18:       218.38 cm**-1
  19:       230.93 cm**-1
  20:       280.51 cm**-1
  21:       281.42 cm**-1
  22:       331.60 cm**-1
  23:       399.03 cm**-1
  24:       467.18 cm**-1
  25:       498.66 cm**-1
  26:       513.31 cm**-1
  27:       571.07 cm**-1
  28:       613.36 cm**-1
  29:       658.26 cm**-1
  30:       671.00 cm**-1
  31:       678.83 cm**-1
  32:       693.12 cm**-1
  33:       694.55 cm**-1
  34:       706.36 cm**-1
  35:       748.03 cm**-1
```

```
36:      749.11 cm**-1
37:      754.29 cm**-1
38:      799.53 cm**-1
39:      836.44 cm**-1
40:      840.56 cm**-1
41:      841.53 cm**-1
42:      844.28 cm**-1
43:      880.07 cm**-1
44:      889.23 cm**-1
45:      979.89 cm**-1
46:     1017.88 cm**-1
47:     1056.16 cm**-1
48:     1130.63 cm**-1
49:     1149.99 cm**-1
50:     1188.63 cm**-1
51:     1197.92 cm**-1
52:     1199.54 cm**-1
53:     1202.36 cm**-1
54:     1271.28 cm**-1
55:     1323.42 cm**-1
56:     1387.79 cm**-1
57:     1388.71 cm**-1
58:     1401.19 cm**-1
59:     1414.79 cm**-1
60:     1423.58 cm**-1
61:     1424.43 cm**-1
62:     1425.78 cm**-1
63:     1459.76 cm**-1
64:     1539.43 cm**-1
65:     1584.00 cm**-1
66:     2016.38 cm**-1
67:     2954.93 cm**-1
68:     2955.41 cm**-1
69:     2955.64 cm**-1
70:     3024.72 cm**-1
71:     3026.47 cm**-1
72:     3026.73 cm**-1
73:     3066.43 cm**-1
74:     3072.94 cm**-1
75:     3081.19 cm**-1
76:     3081.98 cm**-1
77:     3082.63 cm**-1
78:     3099.89 cm**-1
79:     3107.26 cm**-1
80:     3111.92 cm**-1
```

[1] C. Schäfer, J. Flick, E. Ronca, P. Narang, and A. Rubio, Shining light on the microscopic resonant mechanism responsible for cavity-mediated chemical reactivity, Nature Communications **13**, 7817 (2022).

[2] F. Neese, Software update: the orca program system, version 4.0, WIREs Computational Molecular Science **8**, e1327 (2018).

[3] Z. Fan, Y. Wang, P. Ying, K. Song, J. Wang, Y. Wang, Z. Zeng, K. Xu, E. Lindgren, J. M. Rahm, A. J. Gabourie, J. Liu, H. Dong, J. Wu, Y. Chen, Z. Zhong, J. Sun, P. Erhart, Y. Su, and T. Ala-Nissila, GPUMD: A package for constructing accurate machine-learned potentials and performing highly efficient atomistic simulations, The Journal of Chemical Physics **157**, 114801 (2022), https://pubs.aip.org/aip/jcp/article-pdf/doi/10.1063/5.0106617/18280994/114801_1_5.0106617.pdf.

[4] A. Grisafi, D. M. Wilkins, G. Csányi, and M. Ceriotti, Symmetry-adapted machine learning for tensorial properties of atomistic systems, Phys. Rev. Lett. **120**, 036002 (2018).

[5] E. Lindgren, M. Rahm, N. Österbacka, P. Rosander, F. Eriksson, E. Fransson, and P. Erhart, calorine: A python package for constructing and sampling neuroevolution potential models, (2023), to be submitted.

[6] J. Flick, H. Appel, M. Ruggenthaler, and A. Rubio, Cavity born-oppenheimer approximation for correlated electron-nuclear-photon systems, Journal of Chemical Theory and Computation **13**, 1616 (2017), pMID: 28277664, http://dx.doi.org/10.1021/acs.jctc.6b01126.

[7] A. Thomas, A. Jayachandran, L. Lethuillier-Karl, R. M. Vergauwe, K. Nagarajan, E. Devaux, C. Genet, J. Moran, and T. W. Ebbesen, Ground state chemistry under vibrational strong coupling: dependence of thermodynamic parameters on the rabi splitting energy, Nanophotonics **9**, 249 (2020).

[8] C. Climent and J. Feist, On the SN2 reactions modified in vibrational strong coupling experiments: reaction mechanisms and vibrational mode assignments, Physical Chemistry Chemical Physics **22**, 23545 (2020).

[9] X. Li, A. Mandal, and P. Huo, Cavity frequency-dependent theory for vibrational polariton chemistry, Nature communications **12**, 1315 (2021).

[10] J. Sun and O. Vendrell, Suppression and enhancement of thermal chemical rates in a cavity, The Journal of Physical Chemistry Letters **13**, 4441 (2022).
